# Efficient coding theory of dynamic attentional modulation

**DOI:** 10.1371/journal.pbio.3001889

**Published:** 2022-12-21

**Authors:** Wiktor Młynarski, Gašper Tkačik

**Affiliations:** Institute of Science and Technology Austria, Klosterneuburg, Austria; Yeshiva University Albert Einstein College of Medicine, UNITED STATES

## Abstract

Activity of sensory neurons is driven not only by external stimuli but also by feedback signals from higher brain areas. Attention is one particularly important internal signal whose presumed role is to modulate sensory representations such that they only encode information currently relevant to the organism at minimal cost. This hypothesis has, however, not yet been expressed in a normative computational framework. Here, by building on normative principles of probabilistic inference and efficient coding, we developed a model of dynamic population coding in the visual cortex. By continuously adapting the sensory code to changing demands of the perceptual observer, an attention-like modulation emerges. This modulation can dramatically reduce the amount of neural activity without deteriorating the accuracy of task-specific inferences. Our results suggest that a range of seemingly disparate cortical phenomena such as intrinsic gain modulation, attention-related tuning modulation, and response variability could be manifestations of the same underlying principles, which combine efficient sensory coding with optimal probabilistic inference in dynamic environments.

## Abstract 2

Activity of sensory neurons is driven not only by external stimuli but also by feedback signals from higher brain areas, such as those related to attention. A theory developed in this study links diverse, seemingly distinct cortical phenomena to established principles of efficient sensory coding and perceptual inference.

## Introduction

Activity of sensory neurons is highly variable, even in response to the same stimulus [1–3]. Key factors contributing to this variability in the visual cortex are top-down feedback signals from high-level visual areas [4–6]. These signals modulate neural responses to external stimuli and are believed to reflect a broad range of internal states, such as goals of the organism and its beliefs about the state of the environment [7–10].

The question of how internal states of the brain could modulate sensory neurons and contribute to variability of neural activity has been addressed by a number of theoretical studies [9,11]. Neural variability in the primary visual cortex has been linked to probabilistic inference and uncertainty of low-level image features [12–14], as well as to hierarchical inference, where sensory representations interact across different levels of visual pathway to represent progressively more abstract features [15–19]. Structured variability in sensory populations could also result from mechanistic constraints on neural circuit dynamics [20,21].

Attention is a particularly relevant internal state known to modulate sensory codes [5]. Its presumed purpose is to allocate finite neural resources to accurately represent stimuli relevant for the task at hand [5,6]. To account for task specificity, attentional processes are traditionally categorized by the task-relevant properties of the stimulus or the environment into, e.g., object-based attention [22–24], spatial attention [25–27], or feature-based attention [28–30]. Attentional processes are known to modulate neural tuning curves [31], receptive fields [32], and individual neuron firing rates [33,34]. Attentional and other modulatory processes can also influence the collective structure of the population activity, reflected in correlation patterns between pairs of neurons [35–38]. Furthermore, fluctuations in the attentional state can contribute to dynamic variability of neural firing that unfolds over long timescales [1,38–40].

Computational theories of attention have interpreted attention-related modulation of sensory neurons as a consequence of probabilistic inference [41–44], slow fluctuations in the brain state [38], or modulation of gain in hierarchical feed-forward pathways [45]. Despite this progress, we currently do not understand how top-down modulation could enable a key putative feature of attentional computations—namely, the efficient use of limited resources by sensory populations to dynamically encode only the task-relevant sensory information.

Here we address this issue by developing a model of dynamic, top-down modulation of sensory codes. A theoretical grounding of our model is provided by a synthesis of two established normative theories of neural computation: probabilistic inference and efficient coding. Probabilistic inference specifies how task-relevant environmental states can be optimally estimated from unreliable sensory signals. Efficient coding specifies how finite neural resources should be allocated to encode these signals. A fusion of these two theories provides a natural framework to study attentional modulation of sensory codes: a process whose presumed purpose is to allocate finite resources to extract features of the stimulus, which are necessary to accurately estimate relevant properties of the environment [46].

Building on these general principles, and by committing to specific assumptions and simplifications, we develop a model of adaptive sensory representations in the visual cortex. The model is optimized to infer the state of a changing environment from dynamic sequences of natural images. To minimize the amount of neural activity used to encode individual stimuli, the model utilizes top-down feedback to dynamically modulate the gain of individual neurons in the sensory population. This modulation gives rise to an “adaptive code”—a sensory representation that is dynamically adapted in a top-down manner to support perceptual inference in a changing environment.

Adaptive codes can be viewed as the next iteration of the efficient coding paradigm, where the neural code is optimized not only to the statistical structure of the incoming stimuli but also to the statistical structure of the perceptual task [47]. In this way, the bits encoded about the stimulus are the meaningful bits that are essential for a given perceptual task, while the task-irrelevant bits are discarded (making adaptive code a lossy compression scheme) to save resources. The adaptive coding model reproduces known properties of neural coding in the visual cortex and generates novel testable predictions about neural correlations and the impact of perceptual uncertainty on the population code. Our results provide a theoretical account of how top-down modulation could contribute to increased efficiency of sensory representations in the visual system.

## Results

**Fig 1:**
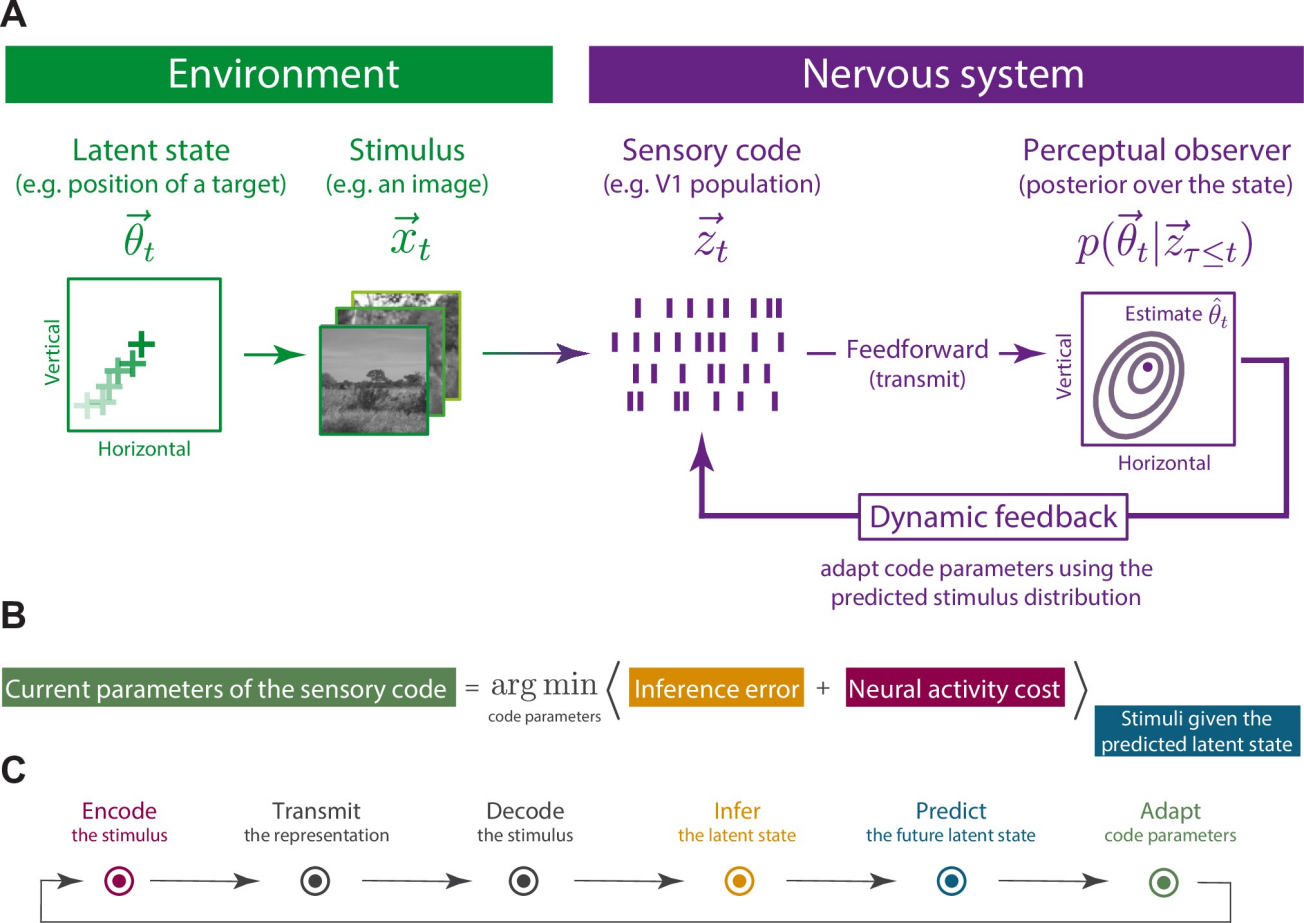
Adaptation of the sensory code for perceptual inference in a dynamic environment. **(A)** Continually evolving state of the environment ${\vec{\theta }}_{t}$ gives rise to a sequence of stimuli ${\vec{x}}_{t}$, which are encoded by a population of sensory neurons into neural responses ${\vec{z}}_{t}$. The properties of sensory neurons (e.g., their gain, receptive fields, recurrent interactions) are not fixed but can be adapted moment by moment via feedback connections from higher brain areas (the model considered here specifically adapts gain of individual neurons). The normative approach we study here considers a scenario where sensory neurons optimally adapt their activation thresholds, leading to maximally accurate inference of the state of the environment by the perceptual observer, at minimal activity cost in the sensory population. Illustrative natural images were taken from [48]. **(B)** Cost function used by the system to adapt the parameters of the sensory code. At each time step, parameters are selected to minimize this cost function. **(C)** A single round of parameter updates consists of multiple steps performed by the sensory system to infer the latent state of the environment from adaptively encoded stimulus stream. Colors correspond to distinct terms of the equation displayed in (B).

We consider a scenario depicted in Fig 1A, where the aim of the sensory system is to keep track of a changing latent state of the environment. This latent state, denoted by ${\vec{\theta }}_{t}$ and evolving in time *t*, might correspond to a behaviorally relevant quantity, such as the position of a moving target. The brain does not have direct access to this latent state and has to infer it from a stream of high-dimensional stimuli ${\vec{x}}_{t}$. Stimuli are encoded by a resource-constrained population of sensory neurons whose instantaneous responses are denoted by ${\vec{z}}_{t}$. A sensory representation of the current stimulus is conveyed via feed-forward connections to a brain region that performs a specific inference (a perceptual observer). To solve this inference optimally, the observer combines the stimulus representation ${\vec{z}}_{t}$ with its internal model of the world into a posterior distribution over the current state of the environment $p({\vec{\theta }}_{t}|{\vec{z}}_{\tau \leq t})$. The posterior distribution is used to extract a point-estimate of the state of the environment ${\hat{\theta }}_{t}$, and the predicted future distribution of stimuli, which we denote as $p({\vec{x}}_{t+1}|{\vec{z}}_{\tau \leq t})$. Based on this prediction, optimal parameters for the sensory population are computed and conveyed back upstream, via feedback connections. These optimal parameters are selected by the perceptual observer to minimize a general cost function schematized in Fig 1B. The cost function navigates a trade-off between two competing objectives: minimization of the expected error in perceptual inference and minimization of the amount of neural activity, which the system requires to encode the incoming stimuli. Parameters of the sensory code are chosen to optimize these two terms, averaged over the stimulus distribution conditioned on the predicted value of the latent state.

Computations described above can be represented as a sequence of steps performed by the model sensory system at each time instant (Fig 1C). By implementing this procedure, the sensory population can use its finite resources to retain only those features of the stimulus, which are relevant to the perceptual observer at any given moment [46], which reflects our intuitions about the role of attention in perception [5].

In the following sections, we develop a model of population coding in the primary visual cortex that implements the general design principles outlined above. We describe first a specific model of neural populations in V1 and endow it with dynamic adaptation whereby the continually evolving perceptual belief adjusts the code to minimize unnecessary neural activity. We then simulate three inference tasks representative of the different kinds of attention studied previously. In the main part of the results, we describe properties of adaptive coding for these tasks and compare them to experimental data.

### Model of adaptive coding in the visual cortex

**Fig 2:**
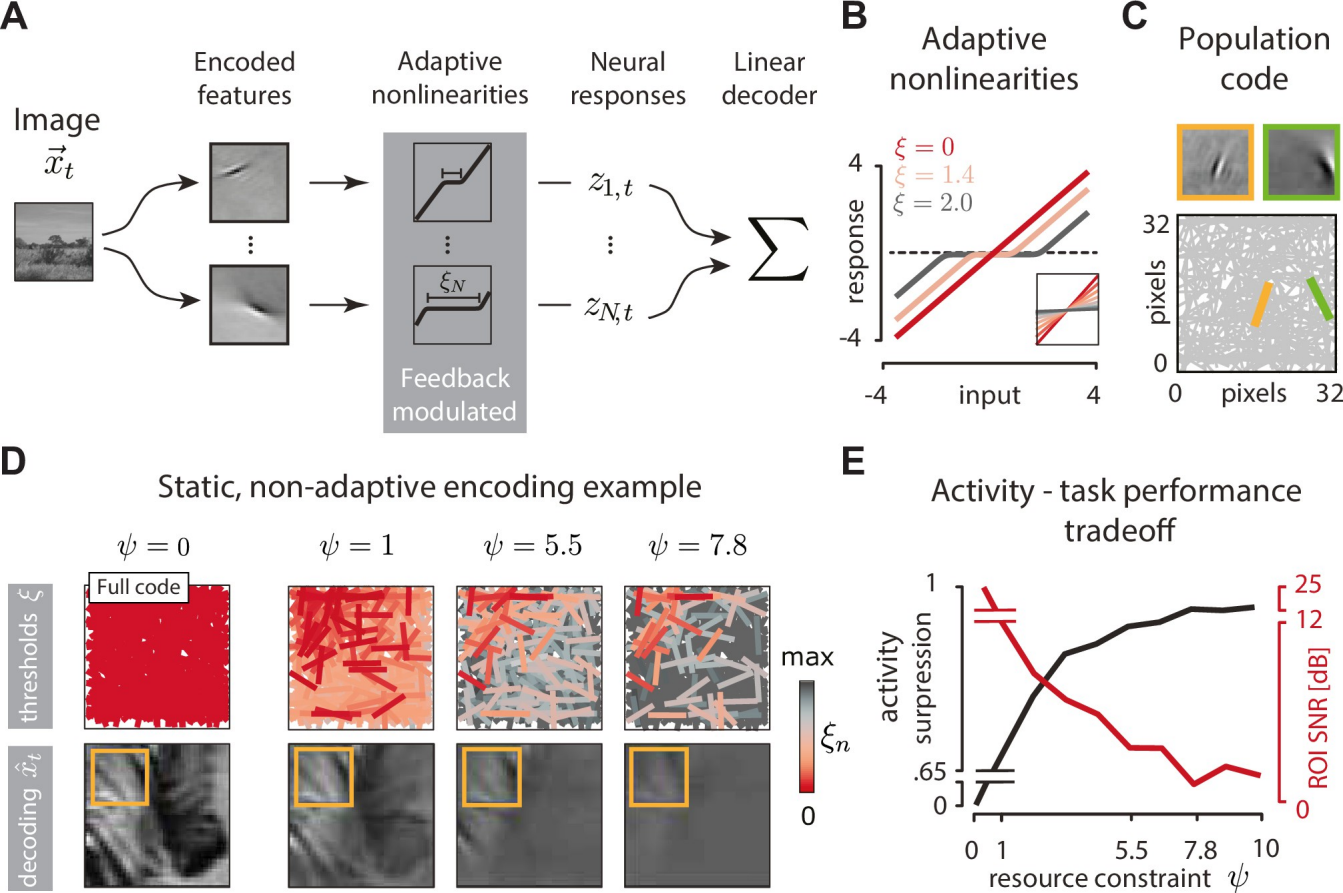
Adaptive population coding with nonlinearities. **(A)** An image ${\vec{x}}_{t}$ (32×32 pixel in size) is encoded by a population of *N* = 512 sparse coding model neurons, characterized by the represented features. Feature activations are transformed by adaptive nonlinearities with threshold parameters *ξ*_*n*,*t*_. The resulting responses *z*_*n*,*t*_ are transmitted to the perceptual observer, which may use them to linearly decode the image and perform further task-specific computations. **(B)** Example adaptive nonlinearities for different values of the threshold parameter *ξ* (color). Inset: linear fits to nonlinearity outputs demonstrate that increasing the threshold *ξ* effectively decreases the neural response gain. **(C)** Visualization of the population code (bottom). The feature encoded by each model neuron is represented by a bar that matches that feature’s orientation and location. Two example features (top) are represented by bars of the corresponding color (bottom). **(D)** Left: an example image reconstructed using the standard sparse code (“full,” when all $\vec{\xi }=0$). Orange frame marks a region of interest (ROI). Right, top row: three sensory populations optimized to reconstruct only the part of the image within the ROI, sorted by increasing attentional resource constraint *ψ*. Red intensity visualizes the value of the optimal thresholds *ξ*_*n*_ (red = low threshold and high gain; gray = high threshold and low gain). Right, bottom row: images linearly decoded from the corresponding sensory populations in the top row. **(E)** Activity of the neural population is increasingly suppressed (black line) and quality of ROI reconstruction (measured in dB SNR) decreases with increasing attentional resource constraint *ψ*.

Following the rationale of Fig 1, we develop a model of adaptive coding in the visual cortex (Fig 2A and 2B), which is an extension of the well-known sparse coding model of V1 [49]. In the sparse coding model, a population of sensory neurons, each encoding a single image feature, forms a distributed representation of natural images. Preferred features of individual neurons are optimized to reconstruct natural images with minimal error, while maximizing the sparsity of neural responses (see Methods). The resulting features resemble receptive fields of V1 neurons and can be conveniently visualized for the entire population [19] (Fig 2C). While sparse encoding is highly nonlinear and requires inhibitory interactions between the neurons [50], images can be linearly decoded from the population activity.

The standard sparse coding model is capable of accurately reconstructing entire images, up to a single pixel, at minimal activity cost. Sparse coding can be viewed as an instantiation of efficient coding of stimuli with a sparse generating structure in a static, task-agnostic setup [51]. We hypothesized that significant further efficiency gains would be possible if the sensory population could dynamically adjust its properties to encode only those image features required by the perceptual observer at any given moment.

We therefore extended the standard sparse coding model by transforming the output of each sparse feature with an adaptive nonlinearity (Fig 2A). Each nonlinearity is controlled by a single parameter *ξ*_*n*_, which corresponds to an activation threshold (Fig 2B). When *ξ*_*n*_ = 0, the response of the neuron *n* is equal to the activation predicted by the standard sparse coding. For *ξ*_*n*_>0, the neuron responds only when the activation exceeds a threshold determined by the value of *ξ*_*n*_. An increase of the threshold can be understood as an effective decrease in the neural gain (Fig 2B, inset). This nonlinear transformation is reminiscent of smooth shrinkage, a well-known image denoising transform [52]. Neural nonlinearities can be dynamically modulated via feedback connections, as we describe more precisely below; what is essential here is that these nonlinearity adjustments allow the resulting neural responses *z*_*t*,*n*_ to be sparsified beyond the standard, task-independent sparse coding. Mathematically, this is achieved by imposing an “attentional resource constraint” of strength *ψ* that penalizes high neural activity ${\vec{z}}_{t}$ (see Eq 1, below). Finally, the neural responses are transferred downstream to the perceptual observer. Image decoding remains a simple, linear transformation.

To illustrate how this model population can selectively encode only the relevant features of a stimulus, we consider a simple, static image encoding task (Fig 2D). We optimize the nonlinearity parameters to reconstruct only a region of interest (ROI) of an image (Fig 2D, orange frame). When the attentional resource constraint is inactive (*ψ* = 0), our model is equivalent to a sparse encoder, and the entire image can be reconstructed with high accuracy (Fig 2D, leftmost column). For increasing values of attentional resource constraint *ψ*, the neuronal thresholds increase and “gain down” neurons that report on the image outside of the ROI (Fig 2D, top row). While the quality of the overall image reconstruction deteriorates with increasing *ψ* (Fig 2D, bottom row), the image within the ROI is preserved with accuracy higher than the rest of the image (which we quantify in signal-to-noise ratio (SNR)). The trade-off between population activity suppression and ROI reconstruction accuracy as a function of the attentional resource constraint *ψ* is clearly visible (Fig 2E). This pedagogical example highlights how task-irrelevant features (here, image components outside of the ROI) can be suppressed in a sensory population to increase coding efficiency. To implement the scenario depicted in Fig 1A, we however need to go beyond a trivial scenario where the system aims to reconstruct a fraction of a static image.

To instantiate adaptive coding, we assume that the perceptual observer dynamically adapts the sensory population via feedback. In order to do so, it sets thresholds of all neurons in the sensory population to optimal values $\xi_{t+1}^{*}$. These values are chosen at every time step *t* to minimize the following cost function:

C(ξ→t+1)=〈DKLsym[p(θ→t+1|z→t+1(ξ→t+1))||p(θ→t+1|z→t+1(ξ→=0))]︸inferenceerrorduetoneuralactivitysuppression+υ∑n=1N|zn,t+1(ξt+1,n)|︸neuralactivitycost〉p(x→t+1|z→τ≤t), (1)

where $D_{KL}^{sym}$ is the symmetrized Kullback–Leibler divergence. We relied on symmetrized variant of the KL divergence because of its conceptual similarity to other error measures such as reconstruction error, but the essence of our framework does not depend on this particular choice.

The cost function in Eq 1 is a concrete instantiation of normative objectives illustrated in Fig 1. The first term corresponds to the error in inference induced by image compression due to suppression of the neural activity via adaptive thresholds (see Methods): This term is small in expectation when the task-relevant predictive information can be retained (at low threshold values). The second term is the neural activity cost, where *ψ* is the attentional resource constraint: This term is small when the predicted neural activations will be sparse (at high threshold values). By minimizing the cost function *C*, the system balances the two opposing objectives and minimizes the error in latent state inference while reducing the amount of neural activity beyond the limit set by standard sparse coding (*ψ* = 0).

To evaluate the cost function in Eq 1, the observer needs to estimate the predictive distribution over future stimuli,

$$p({\vec{x}}_{t+1}|{\vec{z}}_{\tau \leq t})=\int d{\vec{\theta }}_{t+1}p({\vec{x}}_{t+1}|{\vec{\theta }}_{t+1})\int d{\vec{\theta }}_{t}p({\vec{\theta }}_{t+1}|{\vec{\theta }}_{t})p({\vec{\theta }}_{t}|{\vec{z}}_{\tau \leq t}).$$ (2)

Therefore, the ability to predict the value of the relevant latent state ${\vec{\theta }}_{t+1}$ and the stimulus distribution $p({\vec{x}}_{t+1}|{\vec{\theta }}_{t+1})$ is a crucial component of forming an efficient and adaptive representation for dynamic perceptual inference. We note that Eq 2 is a simplification. In real-world scenarios, stimuli ${\vec{x}}_{t+1}$ will depend on additional factors, other than the relevant latent state ${\vec{\theta }}_{t+1}$, and these factors might be correlated in time.

While our approach is grounded in abstract and general theoretical notions captured in substrate-independent terms of the cost function in Eq 1, our model relies on specific choices such as the parametrization of neural gain functions or individual V1 neuron responses. While these choices are clearly important for biological realism of the model, we do not consider them as crucial for the main results of this study, which are largely independent of modeling details. The question of how realistic neural circuits could implement or approximate the required computations is clearly important, but beyond the scope of present work.

### Perceptual inference tasks

**Fig 3:**
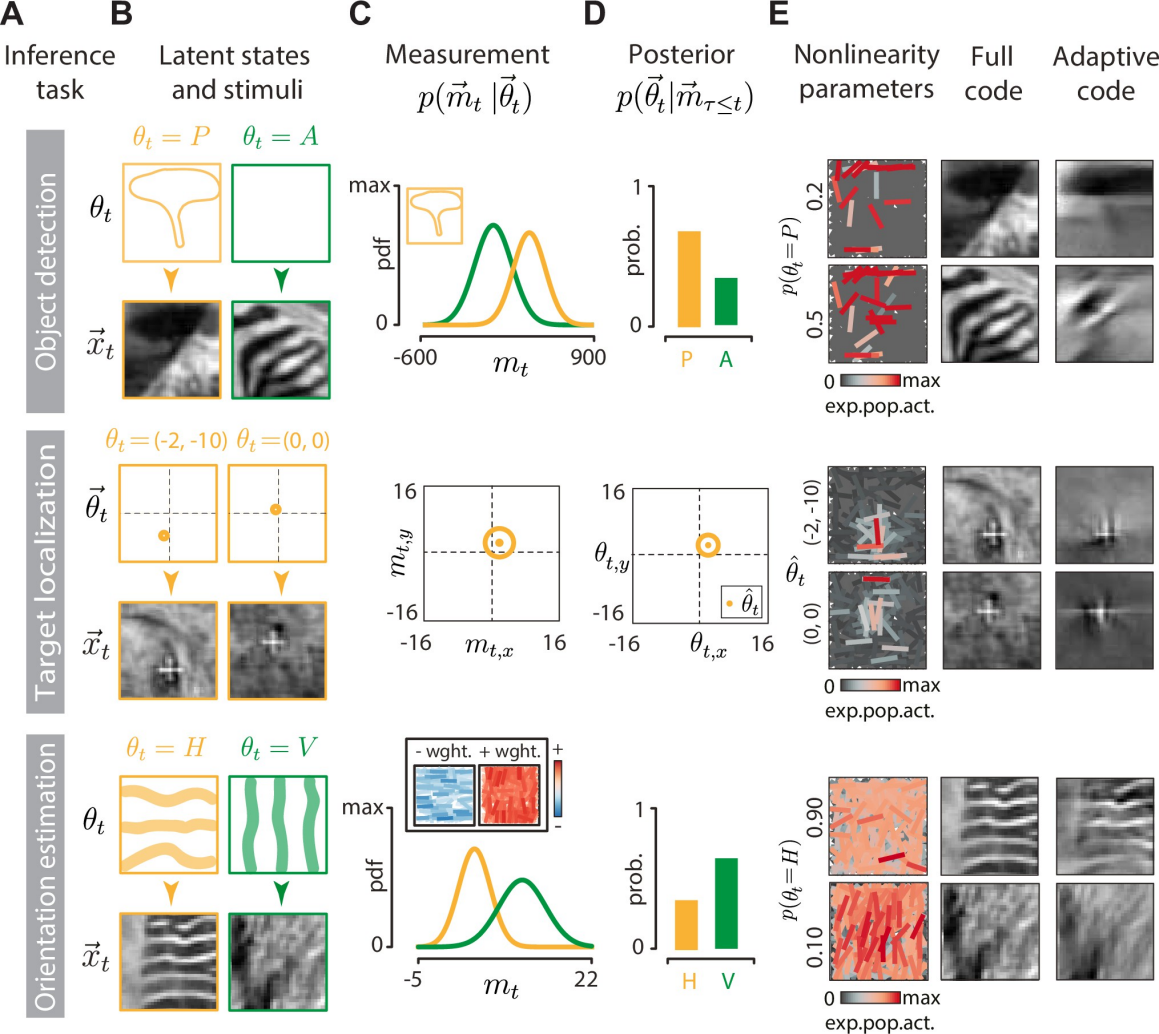
Perceptual inference tasks. **(A)** Rows correspond to individual inference tasks: object detection (top), target localization (middle), and orientation estimation (bottom). **(B)** Visualization of latent states ${\vec{\theta }}_{t}$ (top row of each panel, orange and green frames) and example stimuli ${\vec{x}}_{t}$ in each task (bottom rows of each panel, black frames). Top: tree present (orange) or absent (green). Middle: different white cross positions (orange dot). Bottom: orientation horizontal (orange) or vertical (green). **(C)** Measurements taken by the perceptual observer to infer the state of the environment. Top: a linear decoding of an image is projected onto a target “tree template” (inset, contour outline of the target image) and noise is added. Measurements with object present (orange) and absent (green) follow different distributions. Middle: a linear decoding of an image is used to take a noisy measurement of the target position (orange dot = position estimate; orange circle = noise standard deviation). Bottom: logarithmically transformed neural activity is projected onto a template (inset, blue and red = negatively and positively weighted neurons, respectively) and noise is added. Measurements of predominantly horizontal (orange) and vertical images (green) follow different distributions. **(D)** Example posterior distributions. Top: probability of object being present (P, orange) or absent (A, green). Middle: probability of the visual target location (orange dot = MAP estimate; orange circle = covariance of the estimate). Bottom: probability of the image being predominantly horizontally (H, orange) or vertically (V, green) oriented. Note that specific values displayed in the panel are illustrative. **(E)** Top row, left column: population activity for two different observer belief levels that the tree is present. Top row, middle column: two images decoded using the full code optimized for image reconstruction. Top row, right column: two images decoded using the adaptive code with the activity shown in the left column. Middle and bottom rows: analogous to the top row, but for target localization and orientation estimation, respectively. Throughout, the neural population is visualized using the expected neural activation (colorbar; see Methods).

We consider three different probabilistic inference tasks that the perceptual observer carries out using the adaptive sensory code: object detection, target localization, and orientation estimation (Fig 3A). These tasks correspond to simple variants of traditionally defined types of attention: object-based attention, spatial attention, and feature-based attention, respectively. Each of these tasks is also a case of dynamic inference of a latent variable—a canonical approach to study sensory computations [53].

For each task, the perceptual observer performs a sequence of computations outlined in Fig 1 at each time step. First, the observer uses a representation of the stimulus in the form of population activity vector ${\vec{z}}_{t}$ to perform a “measurement” ${\vec{m}}_{t}$ of the stimulus feature required to infer the latent variable of interest. We introduce the measurement to reflect the fact that the latent state of interest typically does not depend on the entire, high-dimensional representation of the stimulus, but rather on a small number (perhaps just one) of its features. For example, the position of a visual target will not depend on fine structure of the background of the image. The measurement *m*_*t*_ is an auxiliary quantity, which simplifies the description of different perceptual inference tasks but is not essential and is thus not included in the general formulation of the problem, depicted in Fig 1A. The measurement consists of evaluating a task-dependent function *f* over the population activity vector, i.e., ${\vec{m}}_{t}=f({\vec{z}}_{t})+\rho$, where *ρ* is additive Gaussian noise. Second, the measurement ${\vec{m}}_{t}$ is used in a Bayesian update step to compute the distribution over the latent state of the environment $p({\vec{\theta }}_{t}|{\vec{m}}_{\tau \leq t})$, and the predictive distribution of future stimuli $p({\vec{x}}_{t+1}|{\vec{z}}_{\tau \leq t})$. Third, the predictive distribution is used to select optimal values for the neural nonlinearities, to be conveyed to the sensory population via top-down feedback (see Methods for details). To identify the best solution achievable by the model we assume that, as in the ideal observer paradigm [54], the system knows the statistical structure of the task being solved.

#### Object detection

The goal of the object detection task is to infer whether a specific object is embedded in the current image or not (Fig 3A and 3B, top row). The latent state of the environment follows a random correlated process to switch between “object present” (*θ* = *P*) and “object absent” (*θ* = *A*). The observer linearly decodes the image ${\hat{x}}_{t}$ and computes the measurement *m*_*t*_ by projecting the decoded image onto the object template. The measurement *m*_*t*_ follows a different distribution, depending on whether the object is present or absent in the scene (Fig 3C, top row). The posterior distribution is characterized by a single number, the probability of object present *p*(*θ* = *P*) (Fig 3D, top row).

#### Target localization

The goal of the target localization task is to infer the position of a moving visual target—a white cross—embedded in the background of a natural movie (Fig 3A and 3B, middle row). The observer linearly decodes the image to extract a noisy measurement of the position of the target, by computing cross-correlation with the target template (Fig 3C, middle row; see Methods). This noisy measurement, combined with observer’s knowledge of the target dynamics, is used to estimate the current position of the target along the two spatial coordinates ${\hat{\theta }}_{t}=({\hat{\theta }}_{x,t},{\hat{\theta }}_{y,t})$ (Fig 3D, middle row). In this task, the observer relies on these point estimates to adapt code parameters $\vec{\xi }$. In a general scenario, these parameters could be adapted to the entire shape of the posterior over the latent variable *θ*.

#### Orientation estimation

The goal of the orientation estimation task is to determine whether the current stimulus is predominantly horizontally or vertically oriented (Fig 3A and 3B, bottom row). These two classes of images were first discovered via unsupervised learning (see Methods). The latent state of the environment follows a random correlated process to switch between “horizontal” (*θ* = *H*) and “vertical” (*θ* = *V*). The observer projects the magnitudes of neural responses $\left|{\vec{z}}_{t}\right|$ onto a discriminative template, without decoding the image first, to obtain the measurement *m*_*t*_ (Fig 3C, bottom row; see Methods for details). The measurement follows different distributions for horizontally and vertically oriented images (Fig 3C, bottom row). The posterior distribution is characterized by a single number, the probability that the environment is in the horizontal state *p*(*θ* = *H*) (Fig 3D, bottom row).

In addition to the perceptual inference task, the primary factor that impacts the sensory representation, neuronal thresholds *ξ* are modulated also by the strength of the attentional resource constraint *ψ* and, crucially, by the time-changing perceptual belief of the observer (Fig 3E). In the object detection task (Fig 3E, top panel), only the neurons that encode the silhouette of the object are modulated, while the rest of the population remains suppressed to minimize activity. When the observer does not believe that the tree is present in the scene (i.e., *p*(*θ* = *P*) is low; Fig 3E, top panel, top row), only a minimal set of neurons remains active, in order to encode the outline of the tree should it suddenly appear. This is evident when comparing the image decoded from the full code with that from the adaptive code: In the latter case, only the shape of the tree is retained while the rest of the image detail is compressed out. When the uncertainty about the presence of the object increases (i.e., *p*(*θ* = *P*) = 0.5), the sensory population must preserve additional image features to support the perceptual task (Fig 3E, top panel, bottom row).

Similar reasoning applies to the orientation estimation task (Fig 3E, bottom panel), where only the neurons encoding the relevant image orientations remain active and modulated by the observer. While the images reconstructed from the adaptive code lose a lot of spatial detail, they retain the global “gist,” which enables the observer to identify their dominant orientation.

The influence of perceptual belief on the sensory encoding is perhaps most clearly apparent in the target localization task (Fig 3E, middle panel). Here, the sensory population encodes only that region of the image where the perceptual observer believes the target is expected to move in the next time step. This task can be seen as a dynamic generalization of the ROI encoding example of Fig 2D. As the target moves, the observer extrapolates this motion into the future and encodes information just sufficient to confirm or rectify its prediction, while suppressing the rest of the image. This results in an attentional phenomenon that closely resembles a moving spatial “spotlight” of high visual acuity.

This specification of inference tasks completes our setup, and we now turn to discussing the properties of the corresponding adaptive codes.

### Adaptive coding enables accurate inference with minimal neural activity

**Fig 4:**
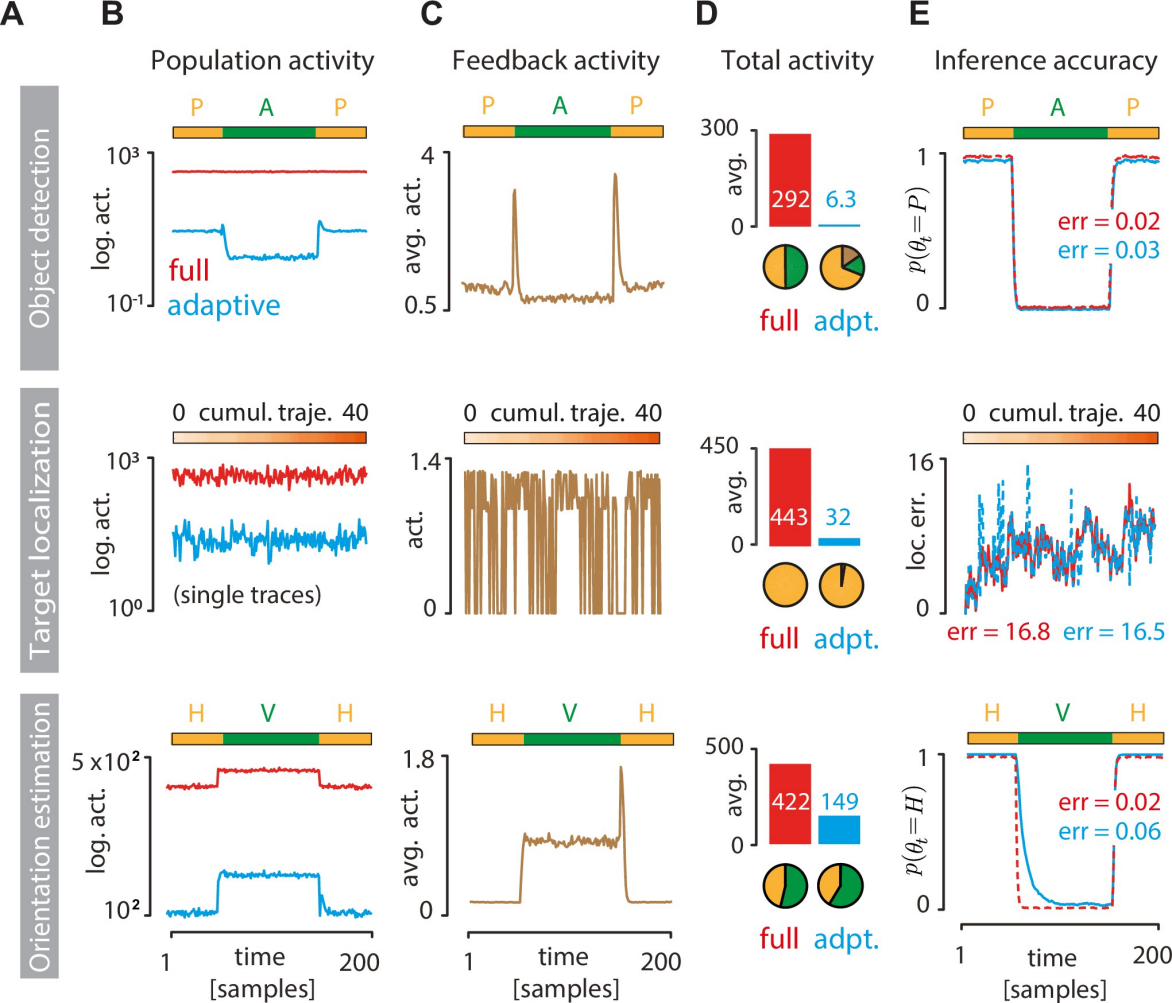
Adaptive coding significantly reduces activity cost with minimal impact on inference accuracy. **(A)** Rows correspond to inference tasks: object detection (top), target localization (middle), and orientation estimation (bottom). **(B)** Sensory population activity 〈|*z*_*n*,*t*_|〉_*n*_ in the standard sparse code optimized for image reconstruction (red = full code) or for a particular task (blue = adaptive code). Activities in object detection (top) and orientation estimation (bottom) tasks were averaged over 500 switches between different states of the environment. For the target localization task (middle), we plot a short nonaveraged activity segment (200 time steps out of a 10^4^ time step simulation; see Methods). **(C)** Same as B but for feedback activity required to adapt the nonlinearities in the sensory population (see Methods). **(D)** Time-averaged activity of the full code (red bars) and adaptive code (blue bars). Pie charts show the total activity decomposed into contributions from two different environmental states (green and orange; top and bottom row only) and feedback (brown; adaptive codes only). **(E)** Inference accuracy (red = full code; blue = adaptive code). Estimates of the environmental state (“object present” in object detection task, top; “orientation horizontal” in orientation estimation task, bottom) were averaged over 100 environmental switches. For the target localization task (middle), inference accuracy is measured as mean squared error between the true and inferred position of the target cross. Text insets display the average inference error in each task (see Methods).

How do adaptive codes navigate the trade-off between minimizing neural activity and maximizing task performance? We simulated perceptual inference in dynamic environments over multiple time steps for all three tasks (Fig 4A). Adaptive coding results in drastic decreases of neural activity in the sensory population compared to the standard sparse coding (Fig 4B). Adaptive coding furthermore reveals interesting task-specific dynamics of population activity, locked to the switches in the environmental state. For example, in the object detection and orientation estimation tasks (Fig 4B, top and bottom panels, respectively), the neural activity is significantly decreased in “absent” and “horizontal” environmental states, respectively. This is because the sensory system needs to extract different kind of information to support downstream inferences in different environmental states. In contrast, the standard sparse code maintains a roughly constant level of activity (Fig 4B, red lines).

We also quantified the cost of top-down feedback signaling (Fig 4C). In our model, feedback activity is commensurate with the amplitude and frequency of posterior belief updates in the perceptual observer (see Methods), making feedback activity patterns strongly task specific. In the object detection task, feedback activity peaks briefly during switches between environmental states (Fig 4C, top panel). In the orientation estimation task, the belief of the perceptual observer fluctuates strongly when vertical orientation dominates, leading to elevated feedback activity (Fig 4C, bottom panel). Since the signal statistics are more homogeneous in the target localization task, feedback activity (when nonzero) stays within a tight interval (Fig 4C, middle panel).

Despite the additional cost of feedback signaling, the total activity of adaptive codes is drastically lower compared to the full sparse code, sometimes by more than an order of magnitude (Fig 4D). This dramatic reduction does not significantly impact the accuracy of the inferences (Fig 4E). Average trajectories of the posterior probability for the object detection and orientation estimation tasks are very similar (Fig 4E, top and bottom panels). In the target localization task, the instantaneous error of the target location estimate using the adaptive code closely follows the error of the full code (Fig 4E, middle panel). For all tasks, the time-averaged error values are comparable between the adaptive and the full code. Taken together, this demonstrates that adaptive coding enables accurate inferences while dramatically minimizing the cost of neural activity in the sensory population.

### Statistical signatures of adaptive coding

**Fig 5:**
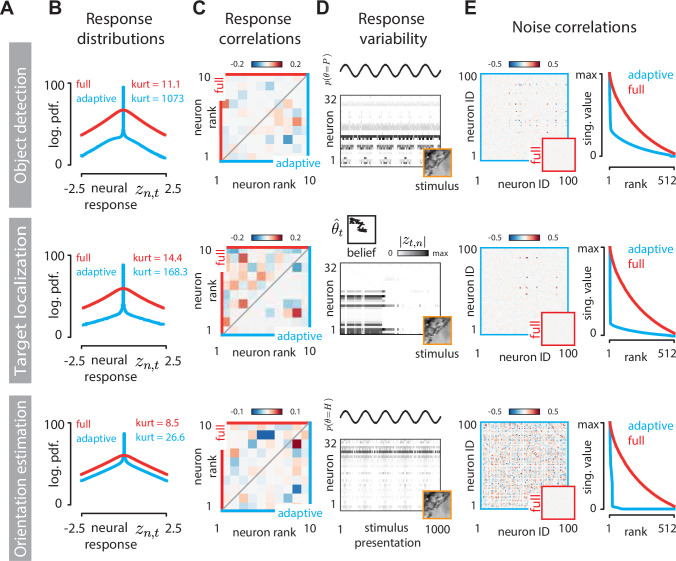
Statistical differences between the adaptive code and the standard sparse code. **(A)** Rows correspond to inference tasks: object detection (top), target localization (middle), and orientation estimation (bottom). **(B)** Distributions of neural responses *z*_*t*,*n*_ for the standard sparse code code optimized for image reconstruction (full, red) and the adaptive code (blue); kurtosis as a measure of sparsness is displayed in inset. **(C)** Pairwise correlations of 10 example neurons whose activity is modulated by the task (different for each task). Correlations were computed over the entire stimulus trajectory used to generate plots in Fig 4. Upper triangle (red) of correlation matrices corresponds to the full code, bottom triangle (blue) to the adaptive code. **(D)** Belief-induced response variability in the adaptive code. Neural activation (grayscale proportional to |*z*_*n*,*t*_|^0.5^) for 32 example neurons chosen separately for each task, exposed to 1,000 presentations of the same stimulus (orange frame). Response variability at fixed stimulus originates from the fluctuations in the internal belief of the perceptual observer (top part of each panel). Here, these fluctuations are simulated as sinusoidal variations in the probability of environmental state (object detection and orientation estimation tasks; top and bottom row, respectively), or a random walk trajectory of the target for the localization task (middle row). **(E)** Belief-induced noise correlations in the adaptive code. Left column: correlation matrices of the same 100 neurons computed from responses to stimulus presentations displayed in (D). Right column: scaled singular values of correlation matrices of the adaptive code (blue). We compared this spectrum to the standard sparse coding in which a small amount of independent Gaussian noise is added to each neural activation. The normalized singular spectrum of noise correlations of the sparse code (red) is denser compared to that of the adaptive code.

Dynamic adaptation significantly changes the statistical structure of a sensory code. The most prominent change is a large increase in the sparsity of the adaptive code compared to the standard sparse code across all tasks (Fig 5A and 5B). This finding is consistent with the observed suppression of average neural activity (Fig 4D). These two phenomena are, however, not exactly equivalent. Sparsity of neural responses (as measured by kurtosis) can be increased in many ways [49], and each would result in suppression of the average activity. In our case, sparsity increase in the adaptive code is induced specifically by a complete suppression of a subpopulation of neurons, resulting in the high spike at zero in the neural response distribution (Fig 5A).

Coordinated top-down modulation of individual neurons leaves its imprint also on the collective statistics of the population activity. For example, different perceptual tasks engage different neurons and, among them, induce different patterns of pairwise correlation. This effect becomes apparent when we focus on a subset of neurons active in a task and compare their correlated activity under standard sparse code or under the adaptive code. In the standard sparse code, neural correlations are inherited solely from the stimulus (Fig 5C, top submatrices, red frame). In an adaptive code, they are additionally modulated by the task, leading to a very different correlation pattern (Fig 5C, bottom submatrices, blue frame).

Changes in the stimulus are not the only factor that drives response variability in the visual cortex. Cortical responses are notoriously unreliable and can fluctuate widely over multiple presentations of the same stimulus [3], giving rise to “noise correlations” among sensory neurons [55–57]. Patterns of noise correlations can be task specific and driven by feedback [37]. Our framework provides a new normative hypothesis about the origin and functional relevance of response variability and noise correlations. In our model, neurons generate different responses even at fixed stimulus when the neural nonlinearities change due to fluctuations in the internal state of the perceptual observer. For example, at the beginning of each target localization trial—even though the stimulus is the same—the perceptual observer may have a different prior belief about where the target is, possibly influenced by preceding history of the neural dynamics or sampling noise that leads to stochastic information accumulation about target position. Trial-to-trial differences in this internal belief will result in a variable allocation of resources in the sensory population as directed by the perceptual observer via top-down feedback, leading to strong noise correlations.

We simulated such a scenario by exposing our model to multiple presentations of a single stimulus, identical across the three tasks, while enabling the perceptual belief to vary. A clear pattern of response variability to multiple presentations of the same stimulus is visible in each case (Fig 5D). This task-specific and feedback-driven response variability manifests in distinct noise correlation structures (Fig 5E, left column). For the adaptive code, the noise correlation matrix is dominated by a small number of modes, reflecting a low-dimensional fluctuating internal state of the perceptual observer. This observation is consistent with the experimentally observed low dimensionality of task-specific correlations in the visual cortex [37,58]. In contrast, noise correlations are expected to be exactly zero for the standard sparse code, within the setting considered here. If independent noise is purposefully introduced into the standard sparse coding units (see Methods), the singular value spectrum is much denser than for the adaptive code (Fig 5E, right column), indicating that the presence low-rank noise correlations differentiates between adaptive and full sparse codes, within the framework described here. In a general setting, noise correlations may be caused by a number of different factors beyond the normative computations described here. For example, they can arise as a consequence of recurrent circuit mechanisms used to compute sparse representations [15,50], or due to the biophysical structure of a neuronal network [21,59–61].

Taken together, adaptive code is predicted to feature: first, a sparser response distribution compared to the standard sparse code; second, task-dependent response correlations compared to task-independent correlations for the standard sparse code; third, prominent yet low-rank noise correlations compared to zero noise correlations for the standard sparse code.

### Adaptive coding reproduces dynamics of internal modulation in the visual cortex

**Fig 6:**
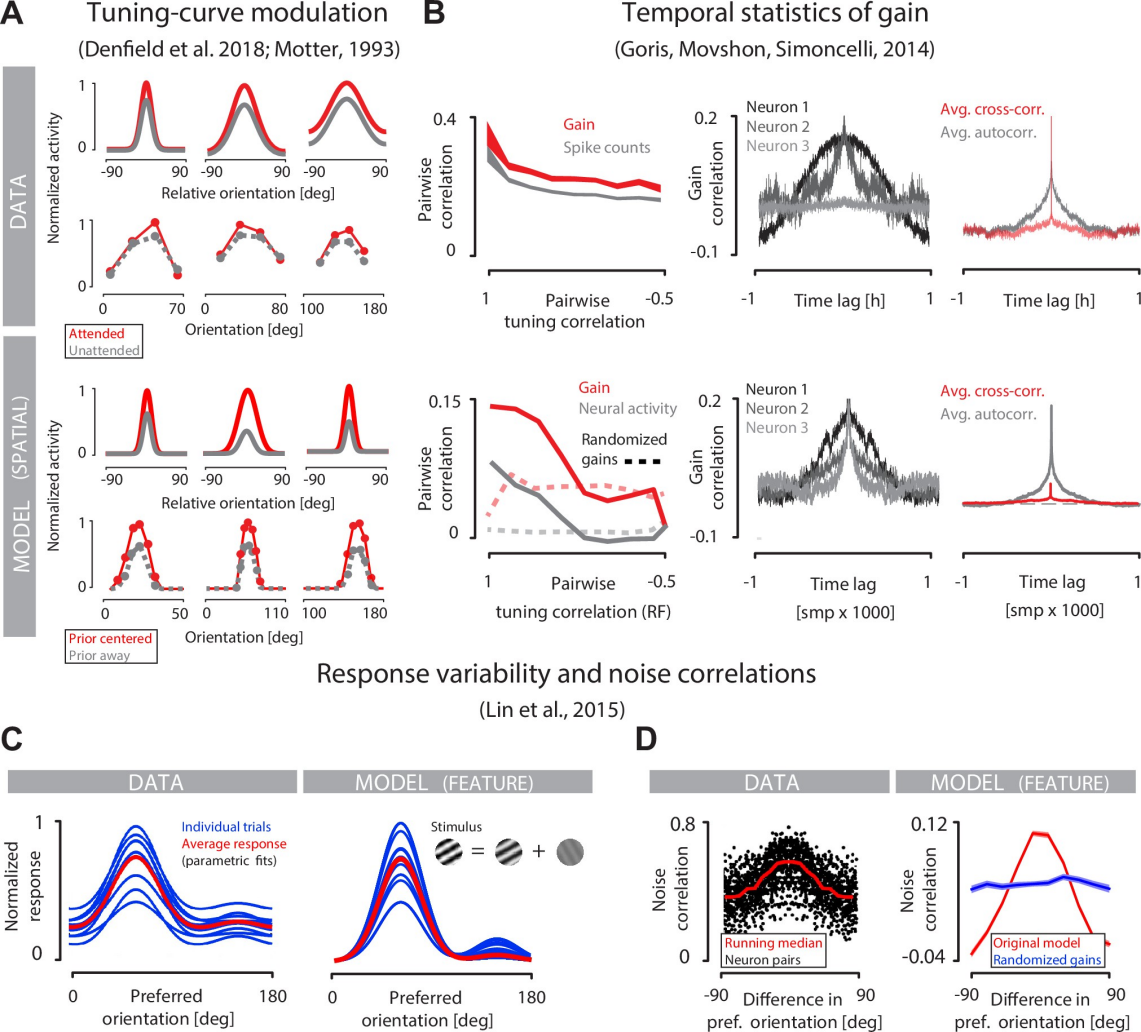
Comparison of adaptive coding model to experimental data. **(A)** Tuning curves of individual neurons in macaque V1 in an attended (red) and unattended (gray) conditions. We display parametric fits to tuning curves centered at the preferred orientation (top panel, top row; replotted from [63]), as well as raw tuning curves (top panel, bottom row; replotted from [62]). Model reproduces the modulation of tuning curves (bottom panel; rows correspond to rows in the top panel; see main text for details). **(B)** Pairwise correlation of internal gain signals (red) and neural activity (gray) as a function of tuning correlation in macaque V1 (top left) is reproduced by the model (bottom left; see main text). Dashed lines denote gain correlations when optimal gain values are randomly reshuffled across the population. Measured gain autocorrelation functions for three example neurons (top middle) span a range of timescales similarly to optimal gain dynamics in the model (bottom middle). Average gain autocorrelation function (gray) and average pairwise gain cross-correlation function (red) are reproduced by the model (data figures—courtesy of Robbe Goris [1] top right; model bottom right). **(C)** Variation of V1 population responses to individual presentations of a mixture of oriented gratings (left, blue lines; data panels in (C) and (D) are reproduced from [65]) fluctuates around the average response (left, red line). Lines depict parametric fits to data. Model optimized for orientation discrimination generates similar pattern of variability (right). **(D)** Noise correlations in V1 depend on the difference in preferred orientation (left, red line denotes the running median). Average noise correlations in the model display similar dependence (right, red line), which disappears after shuffling of neural gains (right, blue line).

To check whether our approach could provide an explanation of experimentally observed phenomena, we compared the properties of the adaptive coding model to three different studies of internal modulation of sensory codes in the primary visual cortex (Fig 6). These studies focus on increasingly complex properties of internally driven modulation of sensory responses in V1: (i) suppression of tuning curves of individual neurons; (ii) statistics of spontaneous gain dynamics; and (iii) coordinated response variability across the entire neural population. Our aim was not to capture the details of any specific experimental setting but rather to verify whether the proposed model could qualitatively account for a broad range of V1 dynamics.

We first focused on the modulation of population tuning curves—a prominent hallmark of spatial attention in the visual cortex [31,62–64]. Orientation-selective neurons whose receptive fields are located in the attended part of the scene respond more strongly to preferred stimuli than neurons encoding unattended parts of the scene (Fig 6A, top panel). This modulation is manifested in the scaling of tuning curves of individual neurons, displayed either as parametric fits (Fig 6A, top panel, top row; reproduced from [63]), as well as raw data (Fig 6A, bottom panel; reproduced from [62]). To simulate such modulation in our model, we relied on the target localization task due to its similarity to the established spatial attention paradigm [5] (Fig 6A, bottom panel). When the perceptual observer expects the target to be present at a particular image location, it increases the gain of neurons reporting on that location, relative to neurons encoding other locations. We interpret this as equivalent to top-down attention being directed towards that location, which allows us to extract from our model a “prior-centered” tuning curve comparable to the “attended” experimental condition. This is to be compared with the “baseline” tuning curve comparable to the “unattended” experimental condition, computed using neural gain averaged over long periods of time (see Methods). We note that this spotlight-like gain modulation was not engineered in any way into our model; instead, it emerged from a generic principle that optimizes perceptual inference under coding cost constraints.

We next focused on response variability in individual neurons, another prominent signature of sensory processing in the visual cortex. This variability can be conveniently separated into sensory drive and gain dynamics [1,39]. Spontaneous gain dynamics could be induced by internal fluctuations of the attentional state [1,38], therefore enabling us to compare gain dynamics to the predictions of our model (Fig 6B). Because changes in effective neural gain are linked to changes in activation thresholds *ξ* in our setup (Fig 2B), we focus on predicted neuron-to-neuron correlations in threshold dynamics as well as individual neuron threshold autocorrelation function (see Methods). Clear similarities emerge. Observed correlations of gain and neural activity decay with decreasing correlation of neuronal tuning, as predicted by our model; furthermore, the activity correlation is consistently lower than the gain correlation, also as predicted (Fig 6B, left column). A broad spectrum of temporal dynamics for the gain of individual neurons is observed in the sensory population: from long temporal correlations to almost instantaneous decay, which is correctly reproduced by our model (Fig 6B, middle column). When averaged over multiple neurons, the gain autocorrelation function shows a smoothly decaying profile. In contrast, the average cross-correlation in gain across pairs of neurons reveals no preferred temporal relationship and decays essentially instantaneously, which is also correctly reproduced by our model (Fig 6B, third column). Further inspection of auto- and cross-correlation functions reveals the origins of this discrepancy. Gain autocorrelations typically decay slowly with time, which is reflected in their average. However, individual cross-correlation functions reveal strong variability and show significant deviations from zero in either positive or negative direction, which cancel each other out during averaging (see S4 Fig). Therefore, the average cross-correlation is not a good representation of cross-correlations of neuron pairs. It remains to be tested experimentally whether gain dynamics in V1 reveal similar statistics.

Third, we analyze how response variability is coordinated across the population, which is reflected in the structure of the noise correlations (Fig 6C). Previous work demonstrated that multiple presentations of the mixture of oriented gratings trigger variable responses across the population of neurons in V1 ([65]; Fig 6C, top-left). In our model optimized for orientation estimation task, the gain of individual neurons is synchronously coordinated to match the perceptual belief via feedback. These belief fluctuations result in population-level variability in the responses reminiscent of V1 dynamics (Fig 6C, bottom left). We note that our model modulates only the gain of individual neurons and therefore cannot capture the baseline firing fluctuations in the V1 data. Nevertheless, it does reveal a qualitatively similar pattern of neuronal variability. Variable stimulus responses in V1 are correlated, and the strength of correlations depends on the difference in preferred tuning (Fig 6D, left). This observation is reproduced by our model specialized for the orientation estimation task (Fig 6D, right). Differences in the absolute magnitude of correlations between experimental data and our model probably imply the existence of additional factors that contribute to shared neural variability, not accounted for by our model.

### New predictions of adaptive coding

Previous theoretical work established a link between perceptual uncertainty about the state of the environment and the influence of stimuli on the perceptual belief [46]. In brief, when a Bayesian perceptual observer is highly certain about the value of a latent state of the environment (strong prior), subsequent sensory signals will only have a small influence over its belief (the posterior will be similar to the prior). In contrast, when the observer is highly uncertain, any individual stimulus can sway the observer’s belief by a large margin (the posterior can differ significantly from the prior). This reasoning leads us to the following hypothesis: Efficient sensory systems gain down stimulus encoding in states of high perceptual certainty and gain up encoding in states of high perceptual uncertainty.

**Fig 7:**
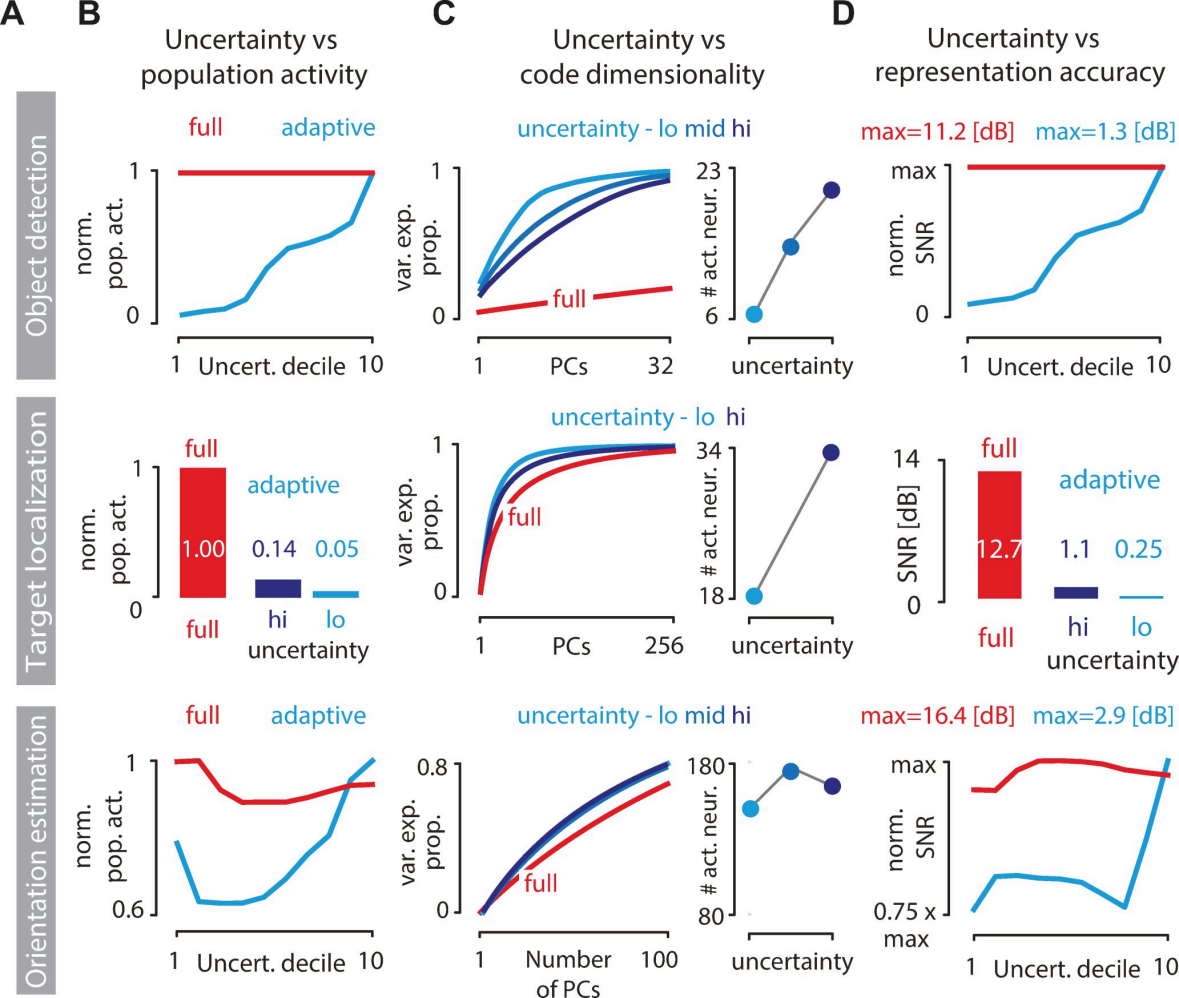
Predicted changes in the adaptive code when perceptual uncertainty is manipulated. **(A)** Rows correspond to inference tasks: object detection (top), target localization (middle), and orientation estimation (bottom). **(B)** Normalized population activity as a function of perceptual uncertainty for the standard sparse code (red = full code) and the adaptive code (blue). Uncertainty in object detection (top) and orientation estimation (bottom) tasks was binned into deciles (see Methods). Uncertainty in the target localization task (middle) is plotted for two levels of measurement noise (dark blue = high noise; light blue = low noise). **(C)** Dimensionality of the adaptive code can increase with increasing perceptual uncertainty (left column). Shown is the proportion of variance in total neural activity explained as a function of the number of principal components (red = full code; light blue = adaptive code at low uncertainty; medium blue = adaptive code at intermediate uncertainty; dark blue = adaptive code at high uncertainty; see Methods). Increase in code dimensionality is correlated with the number of active neurons at different levels of uncertainty (right column). **(D)** Same as (B) but showing the normalized SNR of the image reconstruction at different perceptual uncertainty levels.

We tested this hypothesis in our model. Across all tasks, increases in perceptual uncertainty lead to increased population activity (Figs 7A and 7B, S1 and S2). In contrast, standard sparse coding is not modulated by uncertainty and maintains its activity at a high baseline required to reconstruct the stimuli in full.

Does perceptual uncertainty affect only the total amount of neural activity or also its statistical structure? To answer this question, we assessed the dimensionality of sensory population activity with principal component analysis (PCA) and analyzed it as a function of the entropy of the prior that the perceptual observer holds about the environmental state (see Methods). We find that progressively uncertain observer can engage increasing numbers of neurons (Fig 7C, right column top and middle panels), which affects the dimensionality of the sensory code. When the observer is highly certain, few principal components suffice to explain the population activity; as perceptual uncertainty grows and progressively more neurons are engaged via top-down feedback, the dimensionality of the code increases but always remains bounded by the dimensionality of the full sparse code (Fig 7C). These changes are mirrored in the accuracy of stimulus reconstruction that can be read out from the sensory population (Fig 7D): As perceptual uncertainty grows, incoming stimuli are increasingly relevant for inference and more sensory resources are deployed to encode the stimuli, leading to improvements in stimulus reconstruction.

These results generate two new experimental predictions. First, the average firing rates and the dimensionality of neural activity in the visual cortex should increase during periods of high perceptual uncertainty about the state of the environment. This could be tested, for example, in the target localization paradigm, by comparing experimental conditions in which the target object follows a more versus less predictable trajectory, or where the target is embedded at a higher versus lower contrast in a structured background. To control for sensory confounds and isolate specific effects of perceptual uncertainty, it should be possible to design stimulus protocols where the perceptual task is always performed with an identical probe stimulus, but where perceptual uncertainty was manipulated by prior exposure to different priming stimuli. A specific signature of increasing perceptual uncertainty, which emerges from our model, and which could be measured experimentally, is an increase variability of gain, measured across trials and neurons (see S3 Fig).

Second, under the additional assumption that nonlinearities can change only due to top-down feedback or that they revert to the full code in the absence of feedback, our results predict that silencing of this signaling should decrease the variability of responses in the sensory population. According to our model, the frequency and strength of top-down feedback activity grows with perceptual uncertainty and the frequency of perceptual belief changes. As a consequence, it should be possible to compare the activity of the intact sensory population with the activity of the sensory population where top-down feedback was interrupted via mechanical, pharmacological, or optogenetic means, under stimulus or task conditions that induce large fluctuations in perceptual uncertainty. Disrupted feedback should decrease variability in the sensory population and stabilize its statistics, consistently with the results of [66].

## Discussion

Variability of sensory responses in the cortex has long been ascribed to fluctuations in internal neural processing [4,7,10]. Top-down attention is a particularly important internal process that enhances representations of task-relevant stimuli, at the expense of irrelevant sensory signals. Numerous theories for the origin and functional relevance of top-down attention have been proposed [43,67–71]. In this work, we suggest that several open questions about attentional modulation of sensory codes—about its phenomenology, its effects on the neural code, and its functional origins—are interrelated and fall within the purview of a single conceptual framework that synthesizes two canonical theories of neural computation: optimal perceptual inference and efficient coding [46,72,73].

To make these ideas concrete, we develop a model of sensory coding in the visual cortex that is applicable to dynamic and nonstationary scenarios. We demonstrate that attention-like phenomena emerge as a consequence of moment-to-moment adaptations in a resource-limited sensory code optimized to efficiently learn about the states of the environment. Such “optimal adaptive coding” reproduces a number of observations previously attributed to attention: emergence of the spatial spotlight, tuning curve modulation, gain dynamics, task dependence of neural correlations, and response variability manifesting as noise correlations. We furthermore suggest that different kinds of attention should not be thought of in terms of distinct computational processes but rather as a natural consequence of universal principles of information processing.

Our framework also bears on a puzzling paradox at the heart of how we understand sensory systems. On the one hand, perception and attention seem to rely on coarse, high-level properties of visual scenes, which are encoded selectively depending on the goals and internal states of the brain [74,75]. On the other hand, neurons in the sensory periphery encode signals at the physical limits of precision, right up to individual photons [76]. Why invest in such precision if the information is subsequently not used to guide perception or behavior? Our model shows that adaptive sensory systems, which possess the ability to accurately encode the entire image with a single pixel accuracy, can also dynamically partition this sensory information into the task-relevant part to be extracted and the task-irrelevant part to be suppressed. Precise sensory representations can thus be maintained at a higher cost only when needed; when they suffice for the task, coarse sensory representations are preferred for their efficiency.

### Relationship to other theoretical frameworks

**Fig 8:**
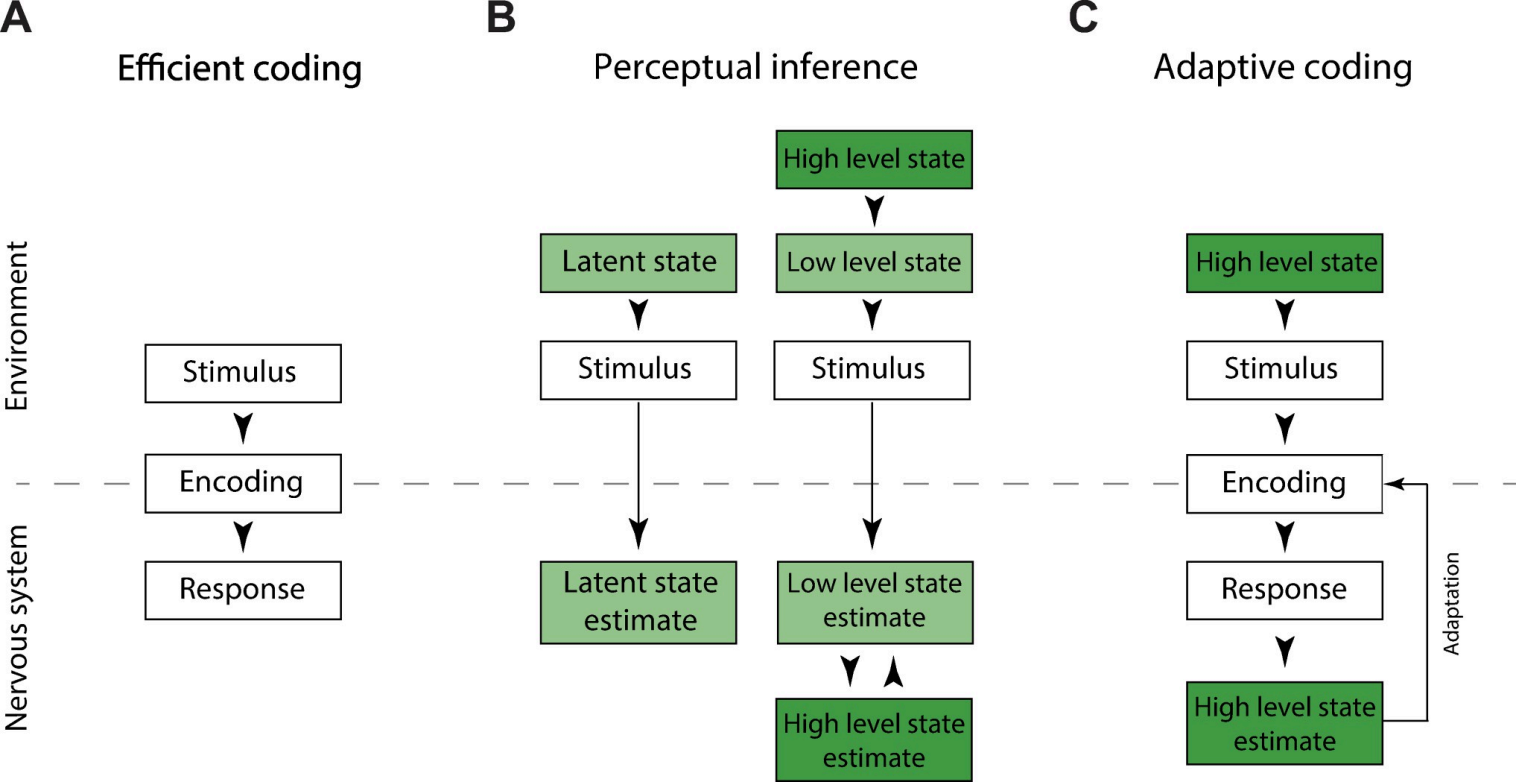
Interpretative frameworks of sensory coding and perceptual inference. **(A)** Normative theories of sensory coding, such as efficient coding, specify encodings (mappings) of low-level stimuli on neural responses. **(B)** Theories of perceptual inference focus on how behaviorally relevant states can be estimated from low-level stimuli (left). Theories of hierarchical inference postulate the existence of a hierarchy of latent states, which are then inferred by the brain from stimuli (right). **(C)** Our approach specifies how an encoding of stimuli can be dynamically adapted, such that only information about task-relevant, high-level latent states is retained by the sensory system, in order to minimize the use of neural resources.

Theories of sensory coding can be broadly categorized by their explanatory scope (Fig 8). For example, the efficient coding framework (first proposed in [77]; Fig 8A) provides a range of normative accounts of how neurons should use their finite metabolic resources to accurately encode either as much stimulus information as possible [49,78] or to encode stimulus features of particular relevance to the organism [47,79,80]. Theories of perceptual inference (Fig 8B, left) place less importance on efficient use of neural resources. Instead, they focus on how the brain could estimate relevant, unobserved (or latent) states of the environment (e.g., position of a predator) from observable stimuli (e.g., retinal images) [54,81,82], and how such computations could be plausibly instantiated (e.g., [83]). Theories of perceptual inference can also take into account the hierarchical organization of the environment (Fig 8B, right), where “high-level” states (e.g., identity of a specific environment) determine statistics of “low-level” sensory information (e.g., local orientation in images). In such settings, the brain is hypothesized to establish a representation that parallels this hierarchical organization of the world [18]. Representations at different levels of such hierarchical systems can interact via multiple feed-forward and feedback information exchanges to establish a complete representation of the stimulus—from abstract, high-level latent states to the low-level image features at individual pixel resolution [16,18,19].

Importantly, theories of efficient coding and perceptual inference are not mutually exclusive [12,73,84] and our model builds precisely on a synthesis of these two theoretical frameworks [46] (Fig 8C). Following perceptual inference approaches, we postulate that the goal of the sensory system is to infer behaviorally relevant, “high-level” latent states from complex and entropy-rich natural stimuli. Following efficient coding approaches, we focus on minimizing the amount of neural resources required to retain information relevant for inference of such “high-level” latent states. Our model exploits the fact that the relevant latent states of the environment are typically low-dimensional and that their estimation may not require representing all the details of the image. For example, to estimate a spatial position of a target, one does not need to accurately encode the details of the background texture. Our model relies on feedback to dynamically compress irrelevant features of stimuli and to retain only the inference-relevant information. This is in stark contrast to theories of hierarchical predictive coding [16], or hierarchical Bayesian inference [18,19] where the top-down feedback provides the values needed for prediction or for explaining away features of the image. In our model, top-down feedback conveys no stimulus information, at least not directly. Instead, feedback conveys the optimal “system settings” for the lossy encoder (e.g., nonlinearity parameters for the sensory population), based on predictions of the perceptual observer. In our scenario, the sensory system does not require multiple feed-forward and feedback passes to establish the stimulus representation. As a consequence, neural resources devoted to coding and time devoted to transmission of sensory information are dramatically reduced. This efficiency comes at a cost: The resulting representation is less robust and unexpected environmental changes may lead to dramatic (but possibly transient) errors in perceptual inference. Examining such errors might provide a viable path to testing the framework of adaptive coding. Taken together, adaptive coding, as instantiated by our model, offers a perspective on the role of top-down feedback in sensory systems that is complementary to previous work.

A key distinction between adaptive coding presented here and the hierarchical predictive coding [16] is that the latter forms a complete representation of the stimulus, from pixel values to high-level latent states; this representation is established across multiple time steps of encoding, transmission, and explaining-away. In contrast, our approach embodies lossy compression that purposefully discards stimulus information, in line with a dynamically evolving internal prediction of the environmental state, task demands, and efficiency constraints. In sum, we are proposing a lossy compression scheme, whereas previous proposals were, in essence, lossless.

A separate class of theories is concerned with how neural circuits may explicitly represent latent variables and associated uncertainty to perform probabilistic inference [12,85–87]. Our model remains agnostic about such neural processes that could be instantiated by the perceptual observer. Instead, we focus on how relevant information can be efficiently extracted from high dimensional stimuli to support estimation of dynamic latent states, regardless of specific inference implementations. Therefore, questions regarding neural representations of uncertainty over latent variables lie outside the explanatory scope of our approach.

Numerous models of top-down attention have been proposed to date [5,70,88,89]. Attention-related changes of sensory representations have been interpreted as a consequence of probabilistic inference [41,42,90], and attention has been postulated as a distinct process that increases gains of neurons relevant to the task [43,45]. In our approach, attention-like processing emerges as a consequence of optimizing a general-purpose objective function. Phenomena such as the spatial spotlight or enhancement of vertical orientations are, therefore, a “side-effect” of this optimization rather than a goal in itself.

To our knowledge, we provide the first theoretical demonstration of how the visual cortex could—and should—perform accurate inferences while dramatically minimizing the cost of neural activity used for stimulus encoding. To date, no work has shown how this frequently postulated yet qualitative rationalization of attention [5,88,91,92] could be instantiated within a mathematical model, for dynamic environments with high-dimensional, natural stimuli. We demonstrate that the response variability, noise correlations, and slow modulations can emerge as automatic consequences of adaptive coding. A salient prediction unique to our model is the relationship between the uncertainty about a high-level, task-relevant latent state (e.g., spatial position of a moving target), and the amount of information about low-level image features present in the neural population, which could be recovered, e.g., via decoding approaches.

Dynamic phenomena such as gain modulation, response variability, and noise correlations are most likely driven by a range of internal processes [93–96]. Empirical dissection of these different factors, and experimental tests of whether the brain relies on computations proposed here, will require coordinated efforts between theory and experiment, which remains a subject of future work.

### Caveats and future work

Our work crucially depends on the observer using the correct statistical model of the environment and its dynamics. Dramatic reduction of neural activity cost with a negligible impact on inference quality cannot be achieved by a “mismatched” observer, which uses an incorrect model of the environment, operates under incorrect assumptions, or fails to correctly compute the optimal thresholds. The question of how such internal model of environmental statistics is learned through evolution and development remains one of the central issues in the field [97].

While our model neural population encodes natural images, perceptual tasks considered here are, at best, naturalistic. Their statistics are designed to easily illustrate the benefits of adaptive coding. Understanding how visual codes can adapt to perceptual tasks that require knowledge of environmental statistics [13,14,54,83] will be a subject of future work.

Our model makes a number of idealizations about the sensory neuron population. Firstly, we assume that adaptive nonlinearities are applied to the output of the sparse coding population, where lateral inhibition plays a crucial role in forming the code [49,50]. Neural firing is computed in a separate step, by transforming these potentials with a thresholding nonlinearity. We envision other possible mechanisms where suppression of unnecessary neural activities occurs simultaneously with the computation of the sparse code, for example, by manipulating sparsity constraints of individual neurons. Secondly, our neural activity is real-valued, making direct quantitative comparisons with spiking data impossible for features such as response variability; this issue could be addressed by extending the model with Poisson spike generation. Furthermore, we make assumptions about the top-down feedback activity. We assume it is instantaneous, whereas real neural circuits may suffer from transmission delays that could detrimentally affect the code performance. We also assume that each change of the parameters of the sensory code is triggered by a single activation of feedback connections. While such strategy would minimize the amount of feedback activity, other mechanisms are possible. For example, following each change, parameters of the code could gradually decay to a baseline value, and sustained feedback activity would be required to maintain them in a desired state [98]. We note that conclusions about the optimality of feedback signalling may depend also on the measure of the feedback cost. The particular measure we adopt here takes into account how many neurons have to be adapted, and how frequently does such change occurs. Other measures may reveal different costs. Lastly, we assume that the brain can precompute and store optimal values of parameters of the sensory code corresponding to different tasks and perceptual beliefs. While optimal, this strategy might be not feasible for neural circuits. A possible approximation strategy would be to store a “basis” of code parameters, which could be flexibly recombined depending on the task at hand, and belief state.

Despite these assumptions, our key insights should not depend on modeling details. Compression of sensory signals could be achieved with different types of nonlinearities, or transformations such as divisive normalization and multiplicative scaling [14,99]. Similarly, stimulus could be represented by alternative schemes, e.g., by neural sampling [12]. Inference carried out by the perceptual observer also need not be explicitly probabilistic [100]. The only essential component of our model is the feedback loop that dynamically adapts the sensory code to the demands of the perceptual observer. This provides the necessary theoretical link between the dynamics of attentional processing, efficient coding, and perceptual inference.

## Methods

### Adaptive coding model of natural images

#### Spare coding model of V1

Standard sparse coding model [49] represents image patches *x*_*t*_ with a population of *N* neurons, each of which encodes strength of a feature ${\vec{\phi }}_{n}$. Given activations of individual neurons *s*_*n*,*t*_, the image patch can be linearly decoded as

x^t=∑n=1Nο→nsn,t. (3)

Basis functions *ϕ* are optimized to jointly minimize the reconstruction error and the cost of neural activity (or, conversely, to maximize sparsity):

$$E(\phi )={\langle \sum_{i}\frac{1}{2\sigma_{SC}^{2}}{\left({\hat{x}}_{t,i}-x_{t,i}\right)}^{2}+\lambda \sum_{n=1}^{N}\left|s_{n,t}\right|\rangle }_{t},$$ (4)

where *λ* is the sparsity constraint, $\sigma_{SC}^{2}$ is the noise level, *i* indexes image pixels, and *t* indexes individual images in the training dataset. We optimized a set of *N* = 512 basis functions using the standard SparseNet algorithm [49], which iteratively alternates between minimizing Eq 4 with respect to basis functions *ϕ* and coefficients *s*. During learning, we fix ||*ϕ*_*n*_||^2^ = 1 for every *n*. To learn neural receptive fields, we used a dataset of 5∙10^4^ 32×32 pixel image patches (standardized to zero mean and unit variance for each patch) randomly drawn from natural movies of the African savannah [101], which were reduced to 512 dimensions using PCA. We learned the sparse features *ϕ* using *λ* = 1 and $\sigma_{SC}^{2}=0.5$; we then fixed features *ϕ* for all subsequent analyses.

#### Adaptive nonlinearities

We extended the sparse coding model by applying pointwise nonlinearities to sparse coding outputs. After encoding an image patch ${\vec{x}}_{t}$, we transformed the activations of individual neurons *s*_*n*,*t*_ into responses *z*_*n*,*t*_:

$$z_{n,t}(s_{n,t};\xi_{n,t},\alpha )=\mathrm{sign}(s_{n,t})\times [\frac{1}{\alpha }\log(\exp(\alpha \xi_{n,t})+\exp(\alpha |s_{n,t}|)-1)-\xi_{n,t}],$$ (5)

where *ξ*_*n*,*t*_ is the threshold value and *α* = 10 is a constant parameter. This nonlinearity is a smooth and differentiable shrinkage operator proposed in [102]. Thresholds *ξ*_*n*,*t*_ are individually set for each neuron at each time point to encode only these features of the image, which are required to perform the perceptual inference.

#### Visualization of nonlinearity parameters

To compare different threshold settings *ξ* in the sensory population across tasks, perceptual beliefs, and stimulus distributions, we visualized the expected neural activity of neuron *n* at time *t*+1: $\langle |z_{n,t+1}|\rangle_{p(x_{t+1}|z_{\tau \leq t})}$. This quantity, which we typically display in color code, would correspond to experimentally observable expected activity of neuron *n*.

#### Cost of feedback activity

We assume that the feedback activity cost at each time point is equal to the standard deviation of the parameter vector ${\vec{\xi }}_{t}$. We computed the cost of feedback activity only at time points *t* when the optimal threshold values changed with respect to time point at *t*−1. The resulting cost measure reflects the frequency of threshold switches and the range of parameter values, which need to be transmitted from the observer to the sensory population via feedback connections after each switch.

### Inference tasks

#### Object detection

**Environment dynamics and stimuli**. At each trial, the environment switches randomly between two states corresponding to two values of the latent variable *θ*_*t*_: object present (*θ*_*t*_ = *P*) and object absent (*θ*_*t*_ = *A*), with the hazard rate *h* = 0.01. When the object was absent, stimuli *x*_*t*_—samples from *p*(*x*_*t*_|*θ*_*t*_ = *A*)—were randomly drawn image patches with zero mean and unit variance. When the object was present, stimuli—samples from $p({\vec{x}}_{t}|\theta =P)$—were a linear combination of a randomly selected image patch ${\vec{x}}_{t}^{R}$, and preselected image of the object of interest ${\vec{x}}_{obj}$ (a tree): ${\vec{x}}_{t}=(1-\gamma ){\vec{x}}_{t}^{R}+\gamma {\vec{x}}_{obj}$, where the mixing coefficient *γ* = 0.2. Sparse coding neural activations *s*_*n*,*t*_ were determined using *λ* = 0.05 and $\sigma_{SC}^{2}=0.5$. We find that higher sparsity values increase the speed of learning the sparse code; however, the precise sparsity value does not have impact on central findings of this work.

**Observer model**. At each time instant *t*, the observer performed the following sequence of steps. First, the observer took the measurement *m*_*t*_ to be a projection of the image reconstructed from the sensory code ${\vec{z}}_{t}$ on the template image of the object of interest ${\vec{x}}_{obj}$, i.e., $m_{t}({\vec{z}}_{t})={\hat{x}}_{t}^{T}{\vec{x}}_{obj}+\zeta$, where *T* is vector transpose and *ζ* is a Gaussian noise with variance $\sigma_{m}^{2}=0.01$. We modelled conditional probabilities *p*(*m*_*t*_|*θ*_*t*_) as Gaussian distributions with class-specific means and standard deviations *μ*_*C*_, *σ*_*C*_ (where *C*∈{*P*, *A*}).

Second, the observer updated the posterior distribution over the latent state *θ*:

$$p(\theta_{t}|m_{\tau \leq t})=\frac{p(m_{t}|\theta_{t})p(\theta_{t}|m_{\tau <t})}{\sum_{\theta_{t}\in \{P,A\}}p(m_{t}|\theta_{t})p(\theta_{t}|m_{\tau <t})}.$$ (6)

From the posterior, the observer computed the MAP estimate, $\hat{\theta }$. For simplicity, we assumed that $p(\theta |{\vec{z}}_{\tau \leq t})=p(\theta |m_{\tau \leq t})$. In the consecutive step, the observer computed the predictive distribution of the latent states $p(\theta_{t+1}|m_{\tau \leq t})=\sum_{\theta \in \{P,A\}}p(\theta_{t+1}|\theta )p(\theta |m_{\tau \leq t})$. At low hazard rate, we could approximate that the predictive distribution is equal to the current posterior, $p(\theta_{t+1}|m_{\tau \leq t})\approx p(\theta_{t}|m_{\tau \leq t})$, from which we derived the predicted distribution of stimuli: $p({\vec{x}}_{t+1}|m_{\tau \leq t})\approx p({\vec{x}}_{t+1}|{\hat{\theta }}_{t})$.

**Nonlinearity optimization**. To avoid the necessity of optimizing nonlinearity parameters at each time step of the simulation, parameters corresponding to different beliefs of the observer were first optimized offline (learned or precomputed). These learned parameters were then used in online simulations. To compute optimal nonlinearity thresholds for sensory encoding at different internal belief states of the observer, we first discretized the posterior distribution over the latent state into *k* = 32 bins, corresponding to linearly spaced values for *p*(*θ*_*t*_ = *P*|*m*_*τ*≤*t*_) over [0,1]. Each of these states defined a distribution of expected image frames, $p({\vec{x}}_{t+1}|m_{\tau \leq t})$. For each of these states, we generated a training dataset consisting of 10^4^ images with and without the object of interest mixed in proportion *p*(*θ*_*t*_ = *P*|*m*_*τ*≤*t*_)/(1−*p*(*θ*_*t*_ = *P*|*m*_*τ*≤*t*_)). For each posterior state, we then numerically optimized the Eq 1 to derive optimal thresholds *ξ* at attentional resource constraint *ψ* = 4, using resilient-backpropagation gradient descent with numerically estimated gradient [103]. Each *ξ* was initialized with Gaussian noise. Since *ξ*_*n*_≥0, we performed the optimization with respect to real-valued auxiliary variables *a*_*n*_, where $\xi_{n}=a_{n}^{2}$. The resulting 32 vectors of optimal nonlinearity parameters ${\vec{\xi }}^{k}$ (where *k*∈{1,…,32}) were used during subsequent simulations, where at each time step the observer selected the most appropriate set of nonlinearities k*:

$$k^{*}=\underset{k}{argmin}[p^{k}-p(\theta_{t}=P|m_{\tau \leq t}){]}^{2},$$ (7)

where $p^{k}\in \{\frac{1}{32},\ldots ,1\}$ is the *k*−*th* discretized value of the belief *p*(*θ*_*t*_ = *P*|*m*_*τ*≤*t*_).

**Simulation details**. We generated a trajectory of the latent states of environment *θ*_*t*_ by concatenating 500 cycles of 50 samples of object present (*θ*_*t*_ = *P*) followed by 100 samples of object absent (*θ*_*t*_ = *A*) and again 50 samples of object present, resulting in the total length of 10^5^ time steps. Analyses in Fig 4B–4E were performed by averaging over the 500 cycles. This artificial environment allowed us to compute averages over multiple changes of the latent state *θ*_*t*_.

#### Target localization

**Environment dynamics and stimuli.** The latent environmental state was defined by the 2D position of the center of the visual target (the white cross 7×7 pixels in size) ${\vec{\theta }}_{t}=(\theta_{t}^{x},\theta_{t}^{y})$, where *θ*_*x*_, *θ*_*y*_∈{1,…,32}. This position evolved as a random walk, $\theta_{t+1}^{C}=\theta_{t}^{C}+\rho$, where $\rho ∼N(0,\sigma^{2})$ and *C*∈{*x*, *y*}; coordinates were rounded to nearest integer and bounded to image dimensions. We chose *σ* = 1.2 for the low-uncertainty scenario and *σ* = 2.4 for the high-uncertainty scenario to analyze the impact of uncertainty on the sensory code. The target was superposed on consecutive frames of a natural movie, ${\vec{x}}_{t}$. Sparse coding neural activations *s*_*n*,*t*_ were determined using *λ* = 0.1 and $\sigma_{SC}^{2}=0.5$.

**Observer model**. The observer computed the measurement ${\vec{m}}_{t}=(m_{t}^{x},m_{t}^{y})$ as the position of the peak of the 2D cross-correlation function between the target template image (the cross) and the stimulus decoded from the neural code ${\hat{x}}_{t}$. We assumed independent measurement noise in spatial coordinates for the measurement *m*_*t*_: $p(m_{t}|\theta_{t})=p(m_{t}^{x}|\theta_{t}^{x})p(m_{t}^{y}|\theta_{t}^{y})$, where marginal conditional distributions of coordinates are Gaussian: $p(m_{t}^{C}|\theta_{t}^{C})=N(\theta_{t}^{C},\sigma_{m}^{2})$ (with *C*∈{*x*, *y*} is the index over spatial coordinates). To simplify optimization, we assumed vanishing measurement noise in this task, *σ*_*m*_ = 10^−5^.

The posterior distribution $p({\vec{\theta }}_{t}|{\vec{m}}_{\tau \leq t})$ can be then computed separately for each spatial coordinate *C*:

$$p(\theta_{t}^{C}|m_{\tau \leq t}^{C})=\frac{p(m_{t}^{C}|\theta_{t}^{C})p(\theta_{t}^{C}|m_{\tau <t}^{C})}{p(m_{t}^{C})}.$$ (8)

The prior distribution $p(\theta_{t}^{C}|m_{\tau <t}^{C})$ and the likelihood $p(m_{t}^{C}|\theta_{t}^{C})$ are Gaussian and conjugate to each other; therefore, the posterior is also Gaussian, $p(\theta_{t}^{C}|m_{\tau \leq t}^{C})=N(\mu_{\theta_{t},C},\sigma_{\theta_{t},C}^{2})$; the point estimate for position is ${\hat{\theta }}_{t}^{C}=\mu_{\theta_{t},C}$. In this scenario, Eq 8 becomes a standard case of Bayesian online estimation of the mean with well-known closed form solutions [104].

We further assume that the observer relies on trivial dynamics, where $p(\theta_{t+1}^{C}|\theta_{t}^{C})=\delta (\theta_{t+1}^{C}-\theta_{t}^{C})$. Therefore the predicted distribution of positions $\theta_{t+1}^{C}$ becomes

$$p(\theta_{t+1}^{C}|z_{\tau \leq t})=\int p(\theta_{t}^{c}|z_{\tau \leq t})p(\theta_{t+1}^{C}|\theta_{t}^{C})d\theta_{t}^{C}=\int p(\theta_{t}^{c}|z_{\tau \leq t})\delta (\theta_{t+1}^{C}-\theta_{t}^{C})d\theta_{t}^{C}=p(\theta_{t}^{C}|z_{\tau \leq_{t}}).$$ (9)

Because the measurement $m_{t+1}^{C}=\theta_{t+1}^{C}+\rho$, where $\rho ∼N(0,\sigma^{2})$, the predicted distribution of measurements along each spatial coordinate is $p(m_{t+1}^{C}|z_{\tau \leq t})\approx N({\hat{\theta }}_{t}^{C},\sigma_{t+1}^{2})$, where the variance is the sum of the variance of the posterior and variance of the random walk, i.e., $\sigma_{t+1}^{2}=\sigma_{\theta_{t},C}^{2}+\sigma^{2}$.

**Nonlinearity optimization**. To compute optimal nonlinearity thresholds for sensory encoding at different internal belief states of the observer, we discretized the posterior belief about the position of the target into 25 values corresponding to a grid of 5 horizontal positions ${\hat{\theta }}_{x}$ and 5 vertical positions ${\hat{\theta }}_{y}$ linearly spaced between 1 and 32 pixels. For each of these positions, we generated a training dataset consisting of 10^3^ images, randomly drawn from a natural image corpus. On each of these images, we superimposed an image of a target (a cross) at a position (*x*, *y*), where each coordinate was drawn randomly from the distribution $N(\mu_{{\hat{\theta }}^{C}},\sigma^{2})$, where *C*∈{*x*, *y*}. For each posterior state corresponding to a spatial position, we then numerically optimized the Eq 1 to derive optimal thresholds *ξ*, using resilient-backpropagation gradient descent with numerically estimated gradient [103]. Each *ξ* was initialized with Gaussian noise. Since *ξ*_*n*_≥0, we performed the optimization with respect to real-valued auxiliary variables *a*_*n*_, where $\xi_{n}=a_{n}^{2}$. The resulting 25 vectors of optimal nonlinearity parameters were used during subsequent simulations. At each time step, the observer selected the optimal nonlinearity vector *ξ*^x*,y*^ corresponding to the discretized position closest to the current position estimate ${\hat{\theta }}_{t}$:

$$(x^{*},y^{*})=\underset{x,y}{argmin}[({\hat{\theta }}_{t}^{x}-x{)}^{2}+({\hat{\theta }}_{t}^{y}-y{)}^{2}].$$ (10)

**Simulation details**. The simulation was ran for 10^4^ steps during which the target trajectory was evolving according to the dynamics described above.

#### Orientation estimation

**Environment dynamics and stimuli**. The environment state *θ*_*t*_ was switching randomly between two states with hazard rate *h* = 0.01. One of the states was generating images dominated by the vertical orientation *θ*_*t*_ = *V* and the other images with predominantly horizontal orientation *θ*_*t*_ = *H*. We identified these two states of the environment via unsupervised learning. First, we used the sparse coding model (without nonlinearities) to encode a large corpus of natural image patches ${\vec{x}}_{t}$. We then transformed activations of each model neuron *n* in response to each patch *t* by taking the log-ratio of its absolute value and the average magnitude of the activation of that neuron: $r_{n,t}=log\frac{\left|s_{n,t}\right|}{\langle |s_{n,t}|\rangle_{t}}$. We then clustered such transformed vectors of the population response *r*_*t*_ into 9 clusters using the standard K-means algorithm. Out of these 9 clusters, we visually selected two. One of these clusters included encodings of image patches where neurons with horizontally oriented basis functions were active stronger than their average. The other cluster included encodings of image patches where the vertically oriented basis functions were activated more strongly than the baseline. We selected these two sets of image patches to be generated by distributions $p({\vec{x}}_{t}|\theta =H)$ and $p({\vec{x}}_{t}|\theta =V)$, respectively. In this task, we used the following parameters of the sparse coding algorithm to encode the images: *λ* = 0.05 and $\sigma_{SC}^{2}=0.5$.

**Observer model**. In this task, the observer did not explicitly decode the image. Instead, it transformed neural activations *z*_*n*,*t*_ by taking their absolute value: *r*_*n*,*t*_ = |*z*_*n*,*t*_|. This vector of activity magnitude ${\vec{r}}_{t}$ was then projected on the discriminative vector $\vec{d}$ to obtain the measurement $m_{t}={\vec{r}}_{t}^{T}\vec{d}+\zeta$, where *T* denotes vector transpose, and *ζ* is a Gaussian measurement noise with variance $\sigma_{m}^{2}=10^{-4}$. The discriminative vector $\vec{d}$ was a linear discriminant optimized to maximize discrimination accuracy between the two clusters of rescaled activity ${\vec{r}}_{t}$ corresponding to the horizontal and vertical states, respectively. We fitted distributions of noisy measurements *p*(*m*_*t*_|*θ*_*t*_) with a Gaussian distribution for each state of the environment separately, i.e., $p(m_{t}|\theta )=N(\mu_{\theta_{t}},\sigma_{\theta_{t}}^{2})$, where *θ*_*t*_∈{*V*, *H*}. The remaining computations were analogous to the object-detection task.

**Nonlinearity optimization**. We computed optimal nonlinearity thresholds for sensory encoding at different internal belief states of the observer in a way analogous to the object detection task. First, we discretized the posterior distribution over the latent state into *k* = 32 bins, corresponding to linearly spaced values for *p*(*θ*_*t*_ = *H*|*m*_*τ*≤*t*_) over [0,1]. Each of these states defined a distribution of expected image frames, $p({\vec{x}}_{t+1}|m_{\tau \leq t})$. For each of these states, we generated a training dataset consisting of 10^4^ images sampled from the vertical and horizontal orientation categories in proportion *p*(*θ*_*t*_ = *H*|*m*_*τ*≤*t*_)/(1−*p*(*θ*_*t*_ = *H*|*m*_*τ*≤*t*_)). For each posterior state, we then numerically optimized the Eq 1 to derive optimal thresholds *ξ* at attentional resource constraint *ψ* = 4, using resilient-backpropagation gradient descent with numerically estimated gradient [103]. Each *ξ* was initialized with Gaussian noise. Since *ξ*_*n*_≥0, we performed the optimization with respect to real-valued auxiliary variables *a*_*n*_, where $\xi_{n}=a_{n}^{2}$. The resulting 32 vectors of optimal nonlinearity parameters ${\vec{\xi }}^{k}$ (where *k*∈{1,…,32}) were used during subsequent simulations, where at each time step the observer selected the most appropriate set of nonlinearities k*:

$$k^{*}=\underset{k}{argmin}[p^{k}-p(\theta_{t}=H|m_{\tau \leq t}){]}^{2}.$$ (11)

**Simulation details**. We generated a trajectory of the latent states of environment *θ*_*t*_ by concatenating 500 cycles of 50 samples of horizontal state (*θ*_*t*_ = *H*) followed by 100 samples of vertical state (*θ*_*t*_ = *V*) and again 50 samples of the horizontal state. Analyses in Fig 4B–4E were performed by averaging over these 500 cycles.

### Computation of code statistics

#### Selection of task-modulated neurons

We sorted neurons according to how strongly they were modulated by the task. As a measure of the task modulation, we took the ratio of the average activity of that neuron in the full sparse code and in the task-specific, adaptive code $\frac{{\bar{z}}_{n}}{{\bar{s}}_{n}}$. To compute activity correlation matrices in Fig 5C, we selected 10 neurons with high modulation values computed in that way.

#### Response variability

To simulate response variability due to feedback modulation of the sensory code (Fig 5D), we encoded the same, randomly selected image patch 1,000 times while the belief of the observer was changing and adapting neural nonlinearities accordingly.

For the object detection and orientation estimation tasks, we took the trajectory of the changing belief (*p*(*θ* = *P*) and *p*(*θ* = *H*), respectively) to be a sine function rescaled to fit in the interval [0.1, 0.9]. Over the 1,000 stimulus presentations, this sinusoid completed five cycles. For the target localization task, we generated an instance of Gaussian walk, which determined the belief of the observer about the location of the target in the scene.

#### Noise correlations

For each task, we estimated noise correlations by computing correlation matrices of neural responses to 1,000 presentations of the same stimulus (see above). To avoid numerical errors we added a Gaussian noise with variance *σ*^2^ = 0.01 to neural responses *z*_*n*,*t*_, after the stimulus has been encoded at each presentation. Correlations of the full code were all approximately equal to 0, since responses to each stimulus presentation were the same.

#### Code dimensionality, population activity, and representation accuracy as a function of perceptual uncertainty

To characterize the dimensionality of the code, we computed PCA of the neural activity matrix *S*, where individual entries *s*_*n*,*t*_ are responses of the n-th neuron at t-th time point. We plotted the cumulative variance explained as a function of the number of principal components. For object detection and orientation estimation tasks, we performed the dimensionality analysis by dividing the neural responses according to the level of uncertainty of the observer and computing PCA on these responses separately. We quantified the uncertainty as the binary entropy of the prior over the latent state (*H*(*p*) = −*p* log_2_(*p*)−(1−*p*) log_2_(1−*p*), where *p* is the probability of the object being present *p*(*θ* = *P*) in the object detection task, and the image orientation being horizontal *p*(*θ* = *H*) in the orientation estimation task. We defined three such intervals of uncertainty: [0, 0.33), [0.33, 0.66), and [0.66, 1] bits. For the target localization task, we run the simulation for two different levels of spatial uncertainty, determined by the variance of the target movements *σ*^2^.

To characterize the amount of population activity, we computed the average absolute value of neural activations |*z*_*n*,*t*_|. The accuracy of representation was computed as the average SNR dB of the image decoding ${\hat{x}}_{t}$, i.e., $20\;{log}_{10}\frac{\sum_{i}x_{t,i}^{2}}{\sum_{i}(x_{t,i}-{\hat{x}}_{t,i}{)}^{2}}$, where *i* indexes the image pixels. For the object detection and orientation estimation tasks, we computed these average quantities for 10 levels of uncertainty spanned by the deciles of the uncertainty distribution. For the target localization task, we computed them for two different levels of spatial uncertainty, determined by the variance of the target movements *σ*^2^.

#### Determination of the number of active neurons

We declared *n*-th neuron to be active at time *t* if the magnitude of its activity exceeded the 1% of its maximal activity, i.e., |*z*_*n*,*t*_|>0.01 max_*t*_(|*z*_*n*,*t*_|). For each time point, we computed the number of active neurons $N_{t}^{act}$ and averaged this number for different levels of uncertainty.

### Comparisons to data

#### Attentional modulation of population tuning curves

To estimate orientation tuning curves of each neuron, we first generated artificial sinusoidal gratings, spanning 32 orientations between 0 and 180 degrees, as well as a range of frequencies and phase values. We encoded them using the sparse coding algorithm and averaged absolute values of responses of each neuron over the range of frequencies and phases to obtain model orientation tuning curves.

We ran a simulation of the target localization task for 10^4^ steps. The two population tuning curves in Fig 6A were computed using different values of nonlinearity thresholds. To compute tuning curves in the absence of attention, for each neuron, we took the nonlinearity threshold value averaged across the entire duration of simulation and estimated the tuning curve in the way described above. To compute the population tuning curve in presence of attention, we took a single nonlinearity threshold value *ξ*_*n*_ corresponding to the belief that the target is closest to the spatial position of the Gabor filter encoded by that neuron and estimated the tuning curve in the way described above.

To obtain parametric fits of tuning curves for data comparison, we first represented each tuning curve as a function of deviation from the preferred orientation (defined as the maximum of that tuning curve). We then fitted such relative-orientation curves with Gaussian distributions multiplied by a scalar value. We display such fits in Fig 6A (bottom panel, top row).

Tuning curves reproduced in Fig 6A from [62] were traced by hand from the original publication.

#### Temporal statistics of gain dynamics

To compute temporal statistics of nonlinearity parameters, we ran a simulation of the target localization task for 10^4^ steps. We note that while we computed temporal correlations of nonlinearity threshold parameters *ξ*_*n*,*t*_, the results do not qualitatively change if we take an inverse of the threshold $\frac{1}{\xi_{n,t}}$, a parameter more directly related to the gain. As a measure of spatial tuning similiarity, we took the correlation of the absolute values of neural basis functions |*ϕ*_*n*_|. We took the absolute value of neural nonlinearity outputs |*z*_*n*,*t*_| as a measure of neural activity level. Auto- and cross-correlation functions were computed using standard methods. To provide baseline for comparison, we randomly reshuffled population responses and gain values across the population after the simulation was completed.

For the analysis displayed in Fig 6, we selected only the neurons whose average activity magnitude 〈|*z*_*n*,*t*_|〉_*t*_ exceeded the 0.01 of the maximal activity average for all neurons in the population. The results do not qualitatively depend on this selection criterion.

To provide a baseline analysis for the dependence of pairwise receptive field correlation and gain and activity correlations (Fig 6B, left column), we randomly reshuffled optimal gain values across neurons prior to the simulation. In that way, each neuron was modulated by gains optimized for a random different neuron through the entire simulation. We then repeated the simulation and analysis described above.

#### Population response variability

We aimed to emulate results obtained in [65] using our model. First, we generated an artificial stimulus by linearly superimposing two visual gratings of 60 and 150 degrees, multiplied by 1 and 0.2, respectively. To simulate fluctuations of the internal belief, we ran a simulation of the orientation estimation task for 10,000 time steps and then extracted trajectory of gains. We encoded the artificial grating stimulus multiple times, while gains were changing according to the previously simulated trajectory. We took the maximum of a tuning curve of each model neuron (estimated in a way described above, with 16 orientations) to be the preferred orientation of that neuron. We computed population responses by averaging responses of individual neurons, grouped according to their preferred orientation into 32 bins spanning the interval between 0 and 180 degrees. Following [65], we fitted each response with a mixture of two Gaussian curves: $r(\phi )=\alpha_{1}N(\mu_{1},\sigma )+\alpha_{2}N(\mu_{2},\sigma )+b$, where *μ*_1_ = 60, *μ*_2_ = 150 are orientations of the gratings used to create the stimulus, *b* is an additive offset, and *σ* was fixed and equal to 0.35. In Fig 6C, left column, we plot these parametric curves fitted to individual trials (blue lines) and to all trials (red line). We display parametric fits to selected population responses computed in that way.

#### Noise correlations

To study the structure of noise correlations, we presented sinusoidal gratings at 12 different orientations linearly spanned on the [0, 180] degree interval. Each of the stimuli was presented 200 times, while the gains of the population were dynamically evolving as described above. We then computed pairwise correlations between all neuron pairs. Each pair was labeled with a difference of preferred orientations, and pairs were grouped into bins linearly spanning the range from −90 to 90 degrees. We then averaged correlations in each bin. To provide a baseline analysis, we ran the simulation with gains randomly reassigned as for Fig 6B and repeated the analyses described above.

## Supporting information

S1 Fig Statistics of uncertainty, population activity, and representational accuracy. (**A**) Object detection task. Left column: full code (red) optimized for image reconstruction; right column: adaptive code (blue) for inference. Top row: uncertainty vs. population activity; bottom row: uncertainty vs. representation accuracy. Each scatter density plot displays 10,000 points. Red, dashed lines depict the linear fit. (**B**) Same as (A) but for the orientation estimation task.
(TIF)

S2 Fig Impact of the attentional constraint *ψ* on uncertainty-activity and uncertainty-accuracy relations in the orientation-estimation task. (**A**) Uncertainty decile vs. normalized population activity (analogous to Fig 7B) for two values of the attentional constraint *ψ*. (**B**) Correlation between uncertainty and population activity as a function of the attentional constraint *ψ*. (**C**) Uncertainty decile vs. encoding accuracy (analogous to Fig 7D) for two values of the attentional constraint *ψ*. (**D**) Correlation between uncertainty and representation accuracy as a function of the attentional constraint *ψ*.
(TIF)

S3 Fig Average time courses of uncertainty and threshold (gain) variance. (**A**) Object detection task. Top: time course of posterior uncertainty (in bits) averaged over 500 switches between the environmental states (marked with a green-orange bar at the top). Bottom: time course of variances of neural thresholds *xi*_*n*,*t*_ averaged over 500 switches between the environmental states and neurons in the population. (**B**) Same as (A) but for the orientation estimation task.
(TIF)

S4 Fig Additional analyses of the gain autocorrelations and cross-correlations for simulations of spatial tracking task in Fig 6B. (**A**) Log-probability histogram of the peaks of gain cross-correlation functions across all pairs of neurons. (**B**) Distribution of extreme (maximal and minimal) values of cross-correlation functions. (**C**) Distribution of decay times of autocorrelation functions of gains of individual neurons. Decay time was determined as the number of time samples after each the autocorrelation dropped below 0.1. (**D**) Distribution of largest autocorrelation values (after excluding the peak at *τ* = 0). (**E**) Example cross-correlation functions of individual pairs of neurons.
(TIF)

## Abbreviations

PCA: principal component analysis
ROI: region of interest
SNR: signal-to-noise ratio
