# Supplementary figures and images for: Efficient coding theory of dynamic attentional modulation
Source: PLoS Biol. 2022 Dec 21;20(12):e3001889. doi: 10.1371/journal.pbio.3001889 (PMC9831638; doi:10.1371/journal.pbio.3001889)

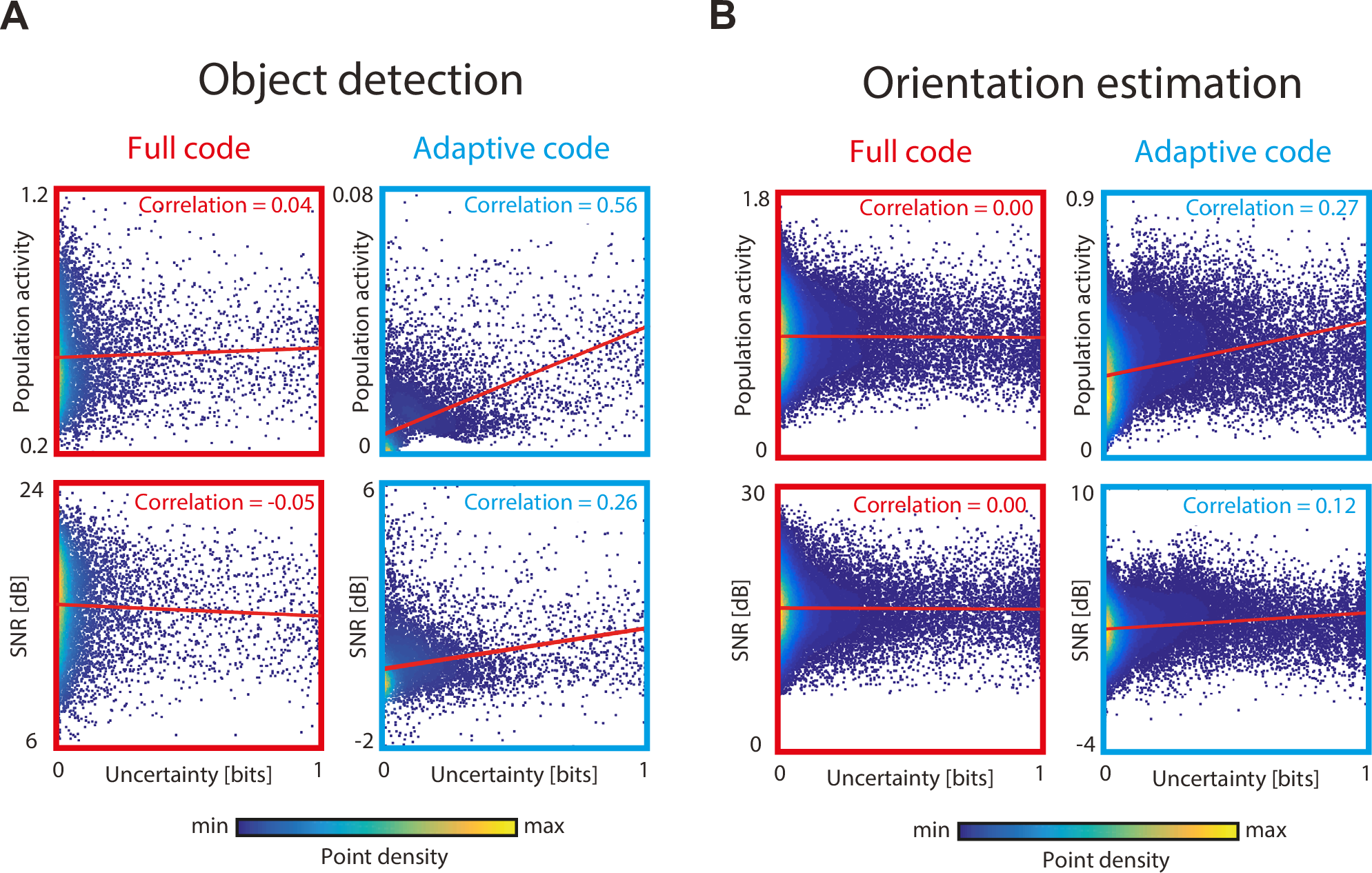

Supplement: S1 Fig — (A) Object detection task. Left column: full code (red) optimized for image reconstruction; right column: adaptive code (blue) for inference. Top row: uncertainty vs. population activity; bottom row: uncertainty vs. representation accuracy. Each scatter density plot displays 10,000 points. Red, dashed lines depict the linear fit. (B) Same as (A) but for the orientation estimation task. (TIF) [file pbio.3001889.s001.tif]

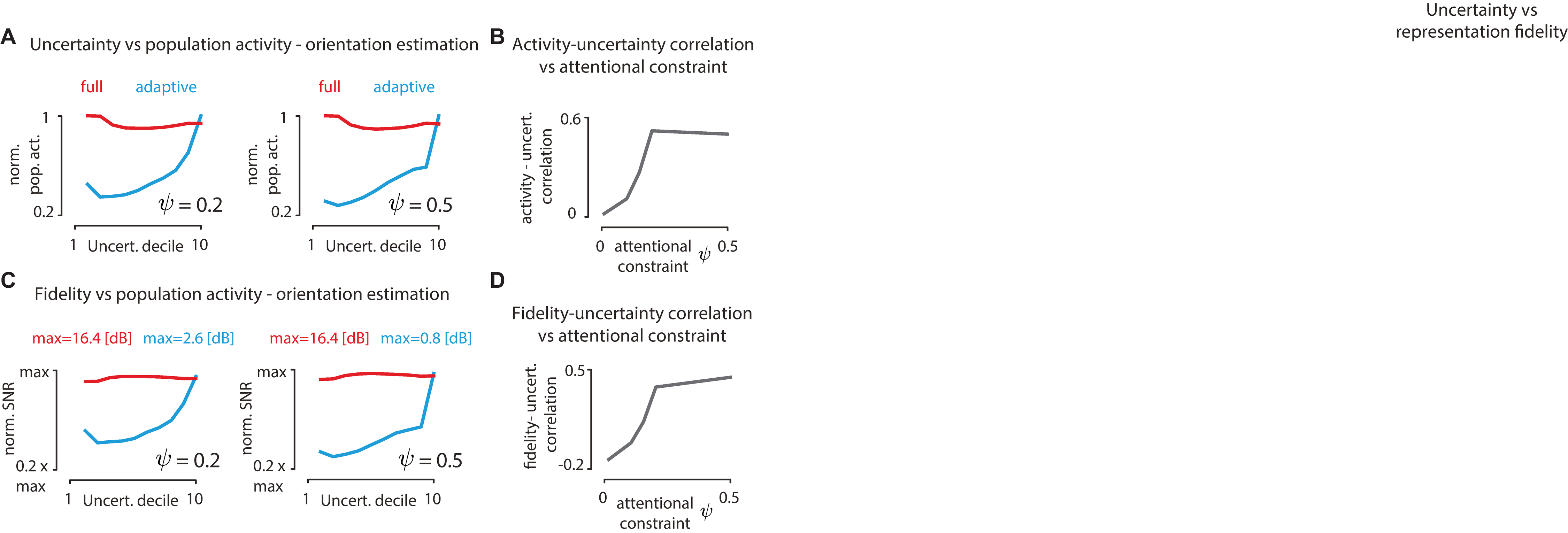

Supplement: S2 Fig — (A) Uncertainty decile vs. normalized population activity (analogous to Fig 7B) for two values of the attentional constraint ψ. (B) Correlation between uncertainty and population activity as a function of the attentional constraint ψ. (C) Uncertainty decile vs. encoding accuracy (analogous to Fig 7D) for two values of the attentional constraint ψ. (D) Correlation between uncertainty and representation accuracy as a function of the attentional constraint ψ. (TIF) [file pbio.3001889.s002.tif]

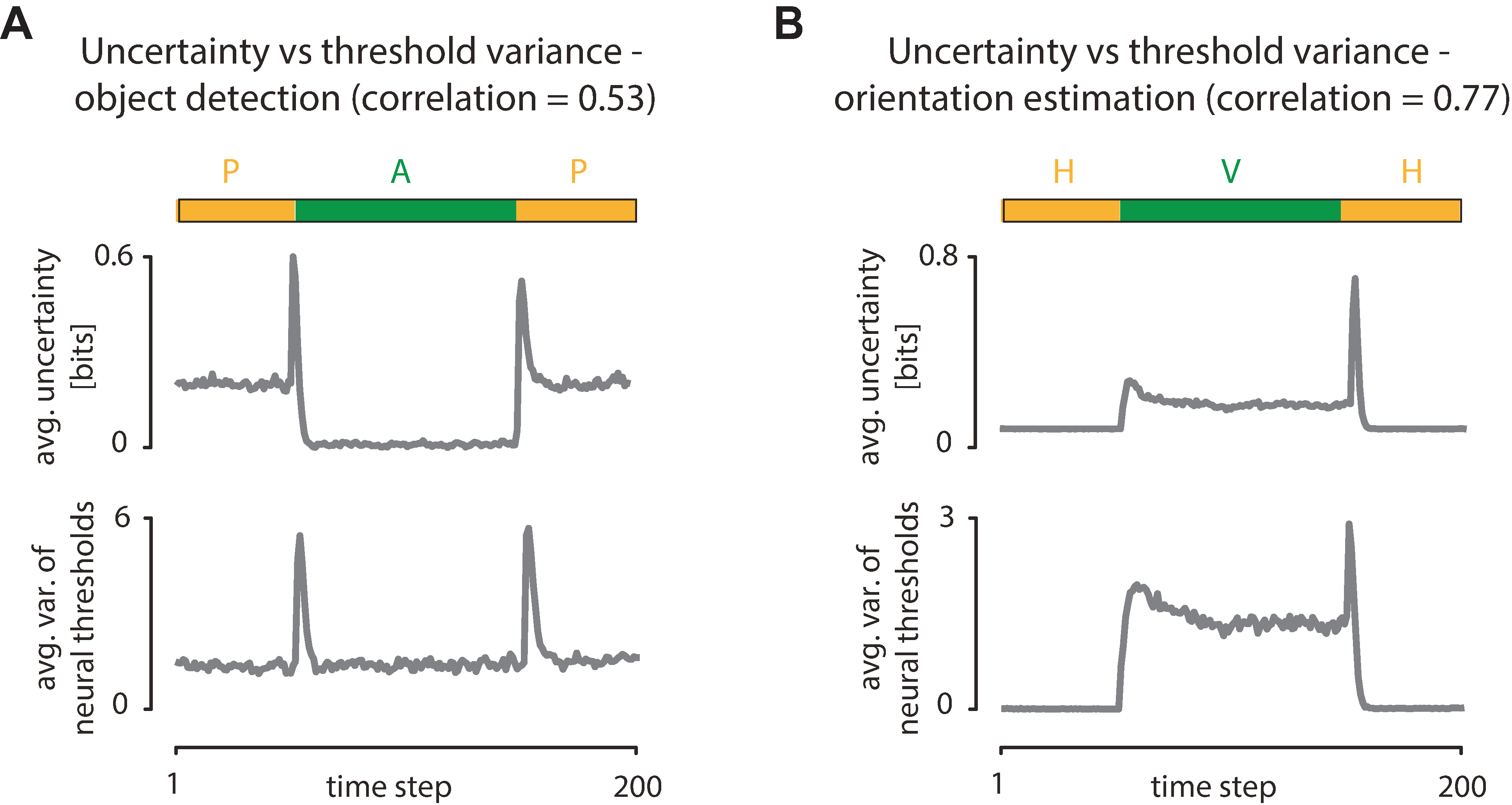

Supplement: S3 Fig — (A) Object detection task. Top: time course of posterior uncertainty (in bits) averaged over 500 switches between the environmental states (marked with a green-orange bar at the top). Bottom: time course of variances of neural thresholds xin,t averaged over 500 switches between the environmental states and neurons in the population. (B) Same as (A) but for the orientation estimation task. (TIF) [file pbio.3001889.s003.tif]

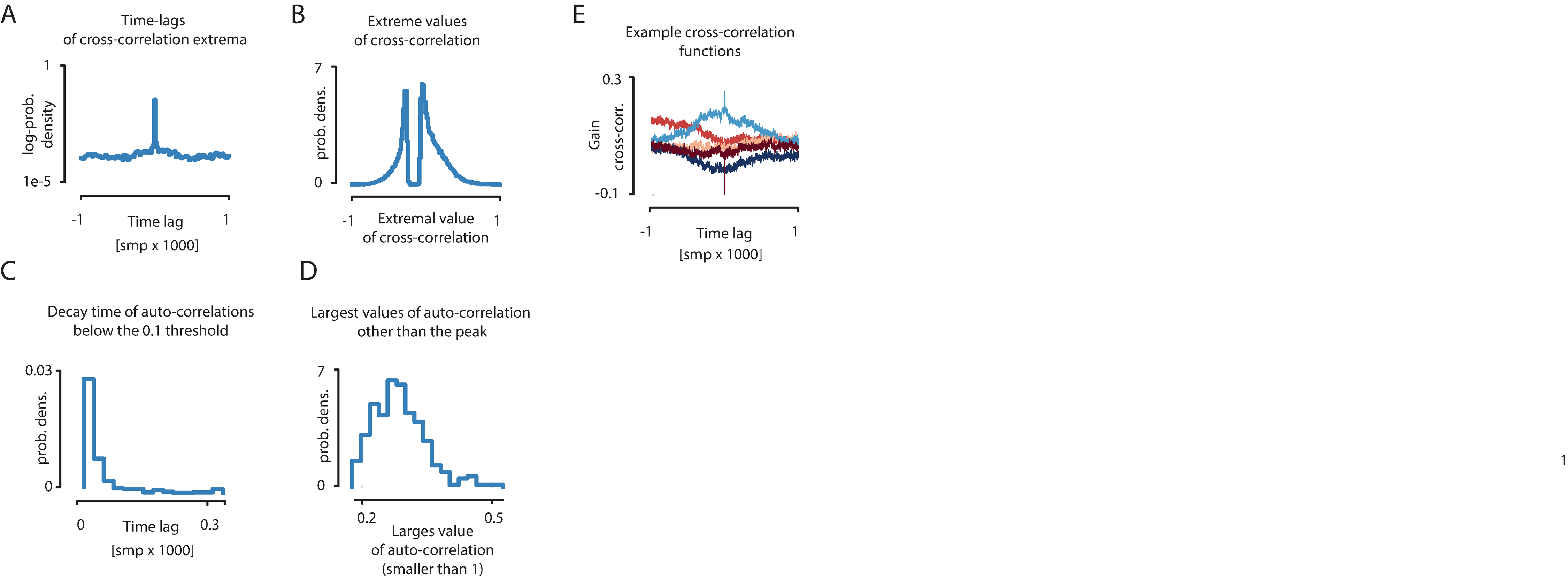

Supplement: S4 Fig — (A) Log-probability histogram of the peaks of gain cross-correlation functions across all pairs of neurons. (B) Distribution of extreme (maximal and minimal) values of cross-correlation functions. (C) Distribution of decay times of autocorrelation functions of gains of individual neurons. Decay time was determined as the number of time samples after each the autocorrelation dropped below 0.1. (D) Distribution of largest autocorrelation values (after excluding the peak at τ = 0). (E) Example cross-correlation functions of individual pairs of neurons. (TIF) [file pbio.3001889.s004.tif]
